# Identification of New Targets and the Virtual Screening of Lignans against Alzheimer's Disease

**DOI:** 10.1155/2020/3098673

**Published:** 2020-08-15

**Authors:** Mayara dos Santos Maia, Gabriela Cristina Soares Rodrigues, Natália Ferreira de Sousa, Marcus Tullius Scotti, Luciana Scotti, Francisco Jaime B. Mendonça-Junior

**Affiliations:** ^1^Laboratory of Cheminformatics, Program of Natural and Synthetic Bioactive Products (PgPNSB), Health Sciences Center, Federal University of Paraíba, João Pessoa, PB, Brazil; ^2^Laboratory of Synthesis and Drug Delivery, State University of Paraíba, João Pessoa, PB, Brazil

## Abstract

Alzheimer's disease (AD) is characterized by the progressive disturbance in cognition and affects approximately 36 million people, worldwide. However, the drugs used to treat this disease are only moderately effective and do not alter the course of the neurodegenerative process. This is because the pathogenesis of AD is mainly associated with oxidative stress, and current drugs only target two enzymes involved in neurotransmission. Therefore, the present study sought to identify potential multitarget compounds for enzymes that are directly or indirectly involved in the oxidative pathway, with minimal side effects, for AD treatment. A set of 159 lignans were submitted to studies of QSAR and molecular docking. A combined analysis was performed, based on ligand and structure, followed by the prediction of absorption, distribution, metabolism, excretion, and toxicity (ADMET) properties. The results showed that the combined analysis was able to select 139 potentially active and multitarget lignans targeting two or more enzymes, among them are c-Jun N-terminal kinase 3 (JNK-3), protein tyrosine phosphatase 1B (PTP1B), nicotinamide adenine dinucleotide phosphate oxidase 1 (NOX1), NADPH quinone oxidoreductase 1 (NQO1), phosphodiesterase 5 (PDE5), nuclear factor erythroid 2-related factor 2 (Nrf2), cycloxygenase 2 (COX-2), and inducible nitric oxide synthase (*i*NOS). The authors conclude that compounds (06) austrobailignan 6, (11) anolignan c, (19) 7-epi-virolin, (64) 6-[(2*R*,3*R*,4*R*,5*R*)-3,4-dimethyl-5-(3,4,5-trimethoxyphenyl)oxolan-2-yl]-4-methoxy-1,3-benzodioxole, (116) ococymosin, and (135) mappiodoinin b have probabilities that confer neuroprotection and antioxidant activity and represent potential alternative AD treatment drugs or prototypes for the development of new drugs with anti-AD properties.

## 1. Introduction

Although Alzheimer's disease is a multifactorial disease [1, 2], it is characterized by the increased generation and/or accumulation of amyloidogenic peptides (particularly A*β*), which are derived from the proteolysis of APP [3]. The presence of senile plaques in the cerebral cortex is thought to result in the activation of inflammatory and neurotoxic processes, culminating in the production of NO, cytokines, and ROS [3–9]. This process contributes to neurodegeneration and the loss of neuronal cells in AD [10, 11].

ROS can have beneficial and negative effects on cellular functions, depending on their concentrations. Low concentrations of ROS can regulate cellular functions, through redox-dependent signaling and redox-dependent transcription factors [8, 9]. However, high concentrations of ROS can impair vital cell processes, causing damage to proteins, lipids, and DNA [10]. Therefore, a balance between the production and removal of ROS is essential for normal cellular functions. Homeostasis imbalances can result in oxidative stress and the subsequent development of pathological conditions [11]. Stress precedes A*β* deposition, tau hyperphosphorylation, and impaired cognitive function. Endogenous antioxidant systems decrease with aging, favoring the appearance of AD. Therefore, oxidative stress is at the heart of AD pathogenesis [12, 13].

Currently, drugs for the treatment of AD include donepezil, galantamine, and rivastigmine, which are inhibitors of the enzyme acetylcholinesterase, while memantine is a noncompetitive inhibitor drug against N-methyl-D-aspartate (NMDA) [14–16]. These inhibitors act on cholinergic receptors and glutamate, respectively. This is because the oxidative glutamate toxicity [13] which is an excitatory neurotransmitter in the central nervous system (CNS) is associated with AD [16]. The excess of glutamate causes the suppression of cysteine uptake by the x_c_^─^ system, which subsequently causes the inhibition of glutathione synthesis (GSH), triggering the accumulation of ROS [17, 18]. In addition to this mechanism, the neurochemical impairment of cholinergic neurons in the central nervous system (CNS) can contribute to the pathology of AD [17]. Although these drugs represent the best pharmacological treatments available at the time of AD, they have a relatively small average overall effect and do not alter the course of the underlying neurodegenerative process [19] probably because AD is multifactorial and is related to several deregulated mechanisms, due to the activation or inactivation of several enzymes important for homeostasis.

Knowing that oxidative stress is the center of the pathogenesis of AD, oxidative defense mechanisms appear to be important targets for the development of new and promising AD drugs. The Kelch-like ECH-associated protein 1 (Keap1)/Nrf2/ARE pathway is one of the most potent defensive systems against oxidative stress [20]. In addition, cyclooxygenase-2 (COX-2), inducible nitric oxide synthase (*i*NOS), NADPH oxidase (NOX), lipoxygenase (LOX), c-Jun N-terminal kinase 3 (JNK-3), protein tyrosine phosphatase 1B (PTP1B), phosphodiesterase type 5 (PDE5), NADPH oxidase, sodium-glucose cotransporter (SGLT)1, SGLT2, and DJ-1 have been associated with the expression of anti-inflammatory mediators, neuroprotection, and ROS regulation and therefore represent promising AD targets [21–29].

Natural products are important alternatives for AD treatment because they contain widely known and reported classes of molecules associated with antioxidant activities, especially polyphenol compounds [23]. Lignans are a class of polyphenol compounds, which, according to Barbosa Filho in Simões (1999) [24], are chemically characterized as dimers formed by the oxidative homocoupling of cinnamic alcohols or the coupling with cinnamic acids.

Drug design is an important strategy in the field of medicinal chemistry, which increasingly requires the use of modern tools to ensure the increased practicality and speed of obtaining results. For example, we often utilize *in silico* studies that seek to understand the properties between a ligand and its respective receptor [25].

### 1.1. c-Jun N-Terminal Kinases (JNKs)

JNKs represent a family of serine-threonine protein kinases that are encoded by 3 genes (JNK1, JNK2, and JNK3) [26]. JNK1 and JNK2 are ubiquitously expressed, whereas JNK3 is primarily expressed in the brain. JNKs are activated by phosphorylation (pJNK), through the activation of mitogen-activated protein (MAP) kinase kinase (MAPK2), by extracellular stimuli, such as ultraviolet light, cytokines, and A*β* peptides [27]. In addition, studies have indicated that JNK can be activated by stress and triggered by harmful external stimuli, via the kinase cascade and oxidative stress, in patients with AD [21]. JNKs are associated with several important functions in the cell, such as inflammation, the regulation of gene expression, cell proliferation, and apoptosis. JNK3 has been implicated in the pathogenesis of AD because JNK3 phosphorylates amyloid precursor protein (APP), which increases the production of A*β* [27]. Due to its fundamental role in neurodegeneration, JNK pathway signaling has been a target for the design of pharmacological and potential therapeutic agents [28].

The activation of the JNK pathway depends on the coordinated interaction among the scaffold proteins that belong to the JNK activation complex, which is capable of mediating signal amplification, ensuring substrate specificity, and coordinating a signaling cascade [29]. Different stimuli can trigger JNK activation, including JNK interaction protein 1a (JIP1a) and JIP1b (also called IB1), JIP2, JIP3 (initially called JSAP1) JNK-associated leucine zipper protein (JLP), and various SRC homology 3 (SH3) domain-containing proteins. Substrates are activated by JNK phosphorylation, mediated by c-Jun, which in turn interact with JunB, JunD, c-Fos, and activating transcription factor (ATF), which constitute the transcription factor activator protein 1 (AP-1), which regulates the maturation of the cellular response to stress and modulates the signals that ultimately lead to the activation of caspases and proteins associated with apoptosis [30, 31].

Studies have found elevated levels of JNK-3 in the brains of living patients with AD compared to levels in controls and that inhibitors kinases, including JNK-3, are able to reduce the effects of neuronal injury induced by A*β* [28, 32–34].

### 1.2. Phosphodiesterases (PDEs)

PDEs represent a group of enzymes, consisting of 11 subtypes (PDE1-PDE11), that control the *c*AMP and *c*GMP hydrolysis rates [31]. Variant PDEs play specific roles in different physiological characteristics and pathological processes. Although most PDE isoforms are expressed in the brain (PDE1, PDE2, PDE3, PDE4, PDE5A, PDE7A, PDE7B, PDE8B, PDE9A, PD10A, and PDE11A), their levels of expression vary among regions [33]. For example, PDE5 and PDE1 are located in the cerebellum, but only in Purkinje neurons; PDE1B is located in subsets of Purkinje cells; PDE6 is restricted to the retina and pineal gland; PDE3B is expressed in proopiomelanocortin and neuropeptide neurons; PDE1 exhibits distribution patterns in the hippocampus, cerebral cortex, thalamus, and striatum [34]; PDE2A is widely expressed in the brain, with the strongest expression in the cortex, striatum, and hippocampus; and PDE4 is widely expressed in the CNS [22].

PDEs can affect neuronal cell survival, and when PDES malfunction, they can play roles in neurodegenerative diseases, such as AD [23]. PDE5 produces anti-inflammatory and neuroprotective effects, increasing NOS expression and *c*GMP accumulation and activating the protein kinase G (PKG) signaling pathway, which plays an important role in the development of several neurodegenerative diseases, including AD, Parkinson's disease (PD), and multiple sclerosis (MS) [24].

During AD pathogenesis, PDE5 hydrolyzes *c*GMP, an important intracellular messenger that activates PKG, triggering a wide range of intracellular signals [25]. The cyclic regulation of AMP/*c*GMP plays a determining role in several memory-related processes because these molecules are critical secondary messengers in the brain that are specifically associated with the memory recovery processes [34]. The levels of these messengers are maintained by the balance between production, catalysis, by adenylyl cyclase and guanylyl cyclase, and degradation, which is mediated by PDEs [35]. PDE5 specifically hydrolyzes *c*GMP [31]. Therefore, PDE5 inhibitors act to increase the levels of *c*GMP in neurons. Age-associated decreases in *c*GMP levels have been related to increased PDE5 expression and activity and the accumulation of A*β* peptide, which inhibits the activation of the NO/*c*GMP pathway [23]. Many studies have shown that PDE5 inhibitors exhibit therapeutic effects on AD by stimulating NO/*c*GMP signaling. PDE5 inhibitors can trigger vasodilation in the brain, resulting in the increased or sustained activation of signaling pathways that impact neuroprotective processes [36]. Therefore, elevating *c*GMP levels through PDE5 inhibition represents an alternative strategy for improving the learning and memory functions of AD patients.

### 1.3. Protein Tyrosine Phosphatase 1B (PTP1B)

PTP1B is a member of the nontransmembrane phosphotyrosine phosphatase family [37] and is a regulator of several processes in the CNS, many of which are therapeutically relevant to AD. Increased PTPB1 activity is associated with insulin deficiency and signaling pathways that are impaired in AD [38]. In addition, increased PTP1B activity can be activated with endoplasmic reticulum neuroinflammation and stress, which are both associated with amyloidosis [36]. The neuroinflammatory response includes the activation of innate immune cells in the brain (microglia), the infiltration of macrophages, and the release of inflammatory mediators, such as NO, cytokines, and chemokines, which are associated with the progression of neurodegenerative diseases [37]. Inflammatory processes and amyloid aggregates have been implicated in neuronal loss and cognitive decline. When activated, PTP1B suppresses many signaling pathways that activate GSK3 and are involved in neurodegeneration.

Trodusquemina is a highly selective PTP1B inhibitor that has been used for the intervention of diabetes and obesity in clinical trials and has been investigated for the selective inhibition of PTP1B in neurons. The results showed that trodusquemina was sufficient to improve spatial learning and memory deficits in hAPP-J20 mice and to prevent the loss of neurons in the hippocampus [39]. In another study, PTP1B expression was found to be regulated by inflammatory stimuli, and PTP1B promotes microglial activation and functions as a critical positive regulator of neuroinflammation [37]. Thus, the inhibition of PTP1B provides a new therapeutic strategy for neuroinflammatory and neurodegenerative diseases.

### 1.4. Nicotinamide Adenine Dinucleotide Phosphate (NADPH) Oxidase (NOX)

NOX is the most studied ROS-generating system [6]. NOX family members are transmembrane proteins that utilize electrons from cytosolic NADPH to reduce oxygen, generating a superoxide anion [16]. Seven known isoforms, NOX1, NOX2, NOX3, NOX4, NOX5, DUOX1, and DUOX2, combined with several subunits to form active enzyme complexes [40, 41]. The only known function of these membrane proteins is the catalysis superoxide anion formation from hydrogen peroxide. Hydrogen peroxide easily permeates cell membranes and can directly damage cells by oxidizing deoxyribonucleic acid (DNA), proteins, and lipids [41].

NOX primarily functions to generate free radicals, and some isoforms can be overregulated by a variety of neurodegenerative factors [41]. Studies have suggested that the genetic and pharmacological inhibition of NOX enzymes may reduce harmful aspects associated with brain injuries and neurodegenerative disorders, resulting in a neuroprotective effect [41]. In particular, the observed lack of benefits associated with various antioxidant strategies may be due to the ineffectiveness of antioxidant molecules *in vivo* or the concomitant attenuation of oxidant regulatory roles [40]. Shimohama et al. [42] reported the translocation of p47phox and p67phox, which strongly suggested that NOX is activated in the AD brain.

Studies with NOX inhibitors exert neuroprotective effects against AD, due to anti-inflammatory properties, through the oligomeric A*β*- (oA*β*-) induced microglial proliferation and the production of proinflammatory factors, including ROS, NO, tumor necrosis factor (TNF)-*α*, and interleukin (IL)-1*β* [42–45].

### 1.5. NADPH Quinone Oxidoreductase 1 (NQO1)

NADPH quinone oxidoreductase 1 (NQO1) is a flavin adenine dinucleotide- (FAD-) dependent cytoplasmic flavoprotein that catalyzes the reduction of two electrons from quinones, quinonimines, and nitroaromatic naphthoquinones and substituted by glutathione, dichlorophenolindophenol (DCPIP) dyes, and an NADPH as an electron donor [12]. Therefore, NQO1, plays a central role in monitoring cellular redox status, protecting against oxidative stress induced by a variety of metabolic situations [44], including the metabolism of quinones and other xenobiotics, through the following mechanisms: (i) functioning as a two-electron donor, to provide a derivation that competes with the formation of ROS; (ii) maintaining reduced coenzyme Q; and (iii) regulating the stress-activated kinase pathway [45].

According to Chhetri et al. [12], the inactivation of the detoxifying enzyme NQO1 has been linked to the progression of AD. Factors that alter NQO1 activity can include genetic predispositions, such as the C690T NQO1 polymorphism, advanced age, cigarette smoking, and various medications [12]. The early expression of NQO1 in astrocytes may reflect a partially protective neuronal cell antioxidant protection system that activates at the beginning of the disease process, whereas the late expression of NQO1 may indicate the delayed activation of this system, as a final attempt to prevent neuronal cell death [46].

The antioxidant activity of NQO1 is essential; however, further studies are necessary to determine whether it should be targeted in the treatment of AD.

### 1.6. Nuclear Factor Erythroid 2-Related Factor 2 (Nrf2)

Nrf2 is a transcription factor that facilitates adaptation and survival under stress by regulating the gene expression of different networks of cytoprotective proteins, including anti-inflammatory and antioxidant proteins and proteins that repair or remove damaged macromolecules [47]. Nrf2 plays a crucial role in maintaining cellular redox homeostasis and regulating the production of ROS by mitochondria. Nrf2 affects changes in the mitochondrial membrane potential (*Δψ*m), ATP synthesis, and lipid peroxidation, and Nrf2 activation under stress conditions or by growth factors can neutralize increases in ROS production by the mitochondria, contributing to neuroprotection [48, 49].

Nrf2 is a key regulator of the body's antioxidant response and is responsible for inducing the expression of genes that encode antioxidant proteins and enzymes, in addition to metabolism detoxification phase II enzymes, which is a critical mechanism associated with cell protection and survival. Nrf2 targets include HO-1, superoxide dismutase (SOD), catalase (CAT), NADPH, NQO1, GSH S transferase (GST), GSH reductase (GR), GSH peroxidase (GPx), thioredoxin (Trx), and glutamate-cysteine ligase (GCL) [50, 51].

In addition to mediating antioxidant and detoxification mechanisms, Nrf2 is responsible for modulating the expression of 200 genes associated with other cellular processes, including the inflammatory response, metabolic regulation, cell proliferation, senescence, and mitochondrial function [52, 53].

Recent studies have investigated the participation of Nrf2, in the mechanisms of apoptosis and neuroprotection associated with Alzheimer's disease and traumatic brain injury, as well as the reduction of the expression of EROs [54].

### 1.7. Sodium-Glucose Transport Protein (SGLT)

Glucose transporters can be divided into two primary families: facilitative glucose transporters (GLUTs) and sodium-dependent glucose cotransporters (SGLTs) [54]. Five primary SGLT isoforms have been identified, SGLT1, SGLT2, SGLT3, SGLT4, and SGLT5; however, SGLT1 and SGLT2, in particular, are associated with the pathways involved in the cellular mechanisms of AD [55].

The SGLT1 isoform is encoded by the SLC5A1 gene and performs glucose transport through a secondary active transport mechanism that uses the Na+ gradient established by the Na+/K+ ATPase pump. This receptor is primarily expressed in the intestine, trachea, heart, testicles, prostate, brain, and kidneys. SGLT1 is characterized as a metabotropic receptor, coupled to transmembrane G proteins, with a secondary structure consisting of 664 amino acid residues, arranged in 14 transmembrane helices with both the NH2 and COOH terminals facing the extracellular side of the plasma membrane. The receptor contains only one N-glycosylation site, at Asn248 [56–58].

The SGLT2 isoform is encoded by the SLC5A2 gene and is found in the kidneys, brain, liver, thyroid, muscle, and heart. The SGLT2 structure is highly similar to that for the SGLT1 receptor and appears to be involved in diabetes and kidney disease mechanisms [54].

Studies have demonstrated the involvement of the factor SGLT1 in Alzheimer's disease, as it is related to cellular mediators of vascular injury [58]. Its activation is associated with a reduction in the levels of epidermal growth factor (EGFR), and its expression can be linked to food and control of insulin release by inhibiting the enzymes *α*-amylase and *α*-glucosidase [59–61].

### 1.8. Factor DJ-1

DJ-1 protein acts as an oxidative stress sensor and eliminates peroxide by self-oxidation [61]. This receptor is also related to cancer pathogenesis and may act as a potential tumor marker [62, 63]. DJ-1 participates in several signaling pathways, including mitochondrial quality control and the reaction to oxidative stress. Cells with high levels of DJ-1 have been shown to be resistant to oxidative stress and neurotoxins, such as 6-OHDA, whereas lower levels of DJ-1 make cells vulnerable to oxidative stress [64, 65].

The DJ-1 receptor was reported to have anti-Parkinson's disease activity, by Dolgacheva and collaborators [66]. The mechanisms addressed included the protection of dopaminergic neurons against neurodegeneration in Parkinson's disease. The authors stated that the wild-type DJ-1 receptor can act as an oxidative stress sensor and as an antioxidant. DJ-1 regulates transcription and protects mitochondria from oxidative stress, in addition to increasing uncoupling protein (UCP)4 and UCP5 levels, which are responsible for mitochondrial decoupling and the consequent decrease in mitochondrial membrane potential. DJ-1 also suppresses the production of EROS and acts on redox factors, such as NF-*κ*B, which acts on anti-inflammatory factors [67].

### 1.9. Cycloxygenase (COX)

Prostaglandins (PGs) are produced by prostaglandin-endoperoxide via synthase/cyclooxygenase (COX), which plays important roles in the etiology and inflammation of autoimmune diseases. COX has 2 isoforms: COX-1, which is permanently expressed in most tissues and organs, and COX-2, which is an inflammation-inducible enzyme that is essential during the inflammation process and in autoimmune disease [68–72]. In addition, COX-2 plays a significant role in aging and skin cancer. PGE2 is a fundamental product of the COX synthesis pathway [70].

COX-2, also known as prostaglandin H synthase 2 (PGHS-2), catalyzes the conversion from arachidonic acid and O_2_ to PGs, which are important lipid mediators involved in numerous physiological aspects and pathophysiological processes. Under normal physiological conditions, COX-2 most often has a low level of expression, but this gene is highly induced in response to inflammation [71–73]. COX-1 is a constitutive enzyme, responsible for maintaining a basic level of PGs, to maintain physiological homeostasis, such as gastrointestinal integrity [73, 74]. COX-1 and COX-2 catalyze the biosynthesis of prostaglandins, prostacyclins, and thromboxanes [68]. COX-1 and COX-2 share a very high degree of sequence identity and very similar active site topologies [75].

Neurodegenerative diseases, such as AD, are sometimes treated with nonsteroidal anti-inflammatory drugs (NSAIDs), which target COX-1 and COX-2 [76].

### 1.10. Nitric Oxide Synthase (NOS)

NOS is formed by a group of three enzymes (*e*NOS, *n*NOS, and *i*NOS), which are responsible for the generation of nitric oxide (NO) from the amino acid *L*-arginine [77, 78]. NO is a free radical gas and is associated with several biological functions, playing key roles in the regulation of blood flow, blood pressure, and oxygen delivery [79–81].

NOS includes endothelial NOS (*e*NOS or NOS1) [81, 82], inducible NOS (*i*NOS or NOS2), and neuronal NOS (*n*NOS or NOS3) [83]. *e*NOS and *n*NOS are characteristically expressed, whereas *i*NOS expression is induced exclusively by appropriate stimuli, such as cytokines, TNF-*α*, infections, chronic inflammation, tumors, interferon *γ*, or hypoxia [83]. During *i*NOS induction, the production of large amounts of NO occurs, in contrast with the other two isoforms [79, 84].

The generalized expression of *i*NOS in the CNS is pathological and is often observed during neurological diseases, such as multiple sclerosis, stroke, and Parkinson's disease [85]. In patients with AD, studies have shown that the number of *i*NOS-positive neurons significantly increases in the brain and is associated with neuronal damage [86].

*e*NOS acts directly on the NO formation rate and acts as a limiting enzyme for this process, based on its expression levels and biological activity [78, 87]. *e*NOS activity also influences the maintenance of vascular and endothelial homeostasis [88–90], in addition to the structure and function of the vascular endothelium [90].

*n*NOS produces NO in both the CNS and the peripheral nervous system, where it acts as a neurotransmitter [91, 92]. Although *n*NOS is the enzyme responsible for NO synthesis in neurons, not all neurons express *n*NOS [93]. However, the excessive activation of *n*NOS can result in neuronal death due to the harmful production of NO [94].

### 1.11. Lipoxygenases (LOXs)

LOXs are a group of dioxygenase enzymes that contain iron and catalyze the stereoselective addition of oxygen to arachidonic acid (AA), docosahexaenoic acid (DHA), and other polyunsaturated fatty acids (PUFAs) [95]. The basic nomenclature of LOXs (except LOX-3) is based on the position of oxygen insertion in a substrate [95, 96]. Five types of LOXs have been identified in mammals, referred to as 5-, 8-, 12-, and 15-LOX and LOX-3 [97, 98].

Although 5-LOX is known primarily as a modulator of oxidation and inflammation [99], according to Chu et al. [100], this pathway can directly influence the pathogenesis of AD. The 5-LOX-*γ*-secretase pathway acts on the formation of A*β* peptides and other molecular diseases, including neuroinflammation, synaptic integrity, and cognitive function, which can contribute to new treatments for AD and associated neurodegenerative problems. High levels of 5-LOX in the nuclear envelope are associated with the release of leukotrienes to attract inflammatory cells [101].

5-LOX is widely distributed in the CNS and has been shown to be positively regulated in the postmortem brain of patients with AD, playing a functional role in the pathogenesis [102], as well as its activation influencing synapses and memory impairment [103]. According to Di Meco et al. [104], 5-LOX is a key enzyme for AD because it is involved in inflammatory responses and is expressed at higher levels in the hippocampi of AD patients compared with healthy adults [105].

Observing that several enzymes are directly and indirectly involved through oxidative stress mechanisms and that their activation and inactivation can contribute to neuroprotection or disease progression, the objective of the research was to explore new targets through virtual screening of lignans to identify molecules with potential anti-AD [106, 107].

## 2. Materials and Methods

### 2.1. Data Collection and Curation

Several enzymes with available biological activity and 3D structure data were selected and investigated in this study. Chemical compounds were selected with known activity against the following enzymes: JNK-3 (CHEMBL2637), PTP1B (CHEMBL335), NFR2 (CHEMBL1075094), NOX1 (CHEMBL1287628), PDE5 (CHEMBL1827), COX-2 (CHEMBL230), and *i*NOS (CHEMBL4EM1). These compounds were used in the bank of images used to construct predictive models (https://www.ebi.ac.uk/chembl/) [108]. The details of the banks can be found in Table 1. The compounds were classified based on the pIC_50_ (−log IC_50_ (mol/l)). The IC_50_ value represents the concentration required for 50% inhibition. However, for the enzyme Nrf2, activation data was used because the activation of this protein would obtain the desired effect. In addition, 159 CHEMBL lignans (Table S1) were assessed by virtual screening to identify molecules with potential activity against enzymes involved in AD progression, according to the workflows presented by Fourches et al. [109]. Three-dimensional structures were generated by ChemaxonStandardiser v.18.17.0, (http://www.chemaxon.org).

### 2.2. Quantitative Structure-Activity Relationship (QSAR) Modeling

The Knime 3.5.3 software (KNIME 3.5.3, Konstanz Information Miner Copyright, 2018, https://www.knime.org) was used to perform the analyses and to generate the *in silico* models. Given the success of our previous studies [110, 111], we opted to perform a 3D QSAR analysis for each bank of enzymes. All studied compounds with a solved chemical structure were saved in special data file (SDF) format and imported into the Dragon 7.0 software [112], to generate descriptors.

The banks of molecules and their calculated descriptors were imported from the Dragon software, and the data were divided into a “Partitioning” tool, using the “Stratified sample” option, which separated the data into Training and Testing sets, which represented 80% and 20% of all compounds, respectively. The sets were randomly selected, but the proportions of active and inactive substances were maintained in both databases.

The Random Forest (RF) algorithm, using WEKA nodes [113], was used to build predictive models. The parameters selected for RF for all models were as follows: the total number of forests was 250, and 1 seed was used for the generation of random numbers. Cross-validation was performed to estimate the predictive power of the developed models.

The external performances of the selected models were analyzed for sensitivity (true-positive rate, or active rate), specificity (true-negative rate, or inactive rate), and accuracy (general predictability). In addition, the sensitivity and specificity of the receiver operating characters (ROC) curve were used because these describe actual performance more clearly than accuracy.

The models were also analyzed using the Matthews correlation coefficient (MCC), which can evaluate the model globally, based on the results obtained in the confusion matrix. The MCC is a correlation coefficient between the observed and predictive binary classifications, resulting in values between -1 and +1, where a coefficient of +1 represents a perfect prediction, 0 represents a random prediction, and -1 indicates the total disagreement between the prediction and the observation [114].

MCC can be calculated using the following formula:

(1) $$\begin{array}{c} \text{MCC}=\frac{\text{VP x VN}-\text{FP x FN}}{\surd \left(\text{VP}+\text{FP}\right)\left(\text{VP}+\text{FN}\right)\left(\text{VN}+\text{FP}\right)\left(\text{VN}+\text{FN}\right)}, \end{array}$$

where VP represents true positives, VN represents true negatives, FP represents false positives, and FN represents false negatives.

The applicability domain (APD) was used to analyze the compounds in the test sets, to evaluate whether the predictions are reliable. The APD is a theoretical chemical space that encompasses the model's descriptors and the modeled response, allowing the estimation of uncertainty when predicting the activity of a compound in the training set used during the development of the model. This technique is important for verifying the reliability of QSAR models by comparing predicted values with observed values [115]. APD is calculated using the following formula:

(2) $$\begin{array}{c} \text{APD}=d+Z\sigma , \end{array}$$

where *d* and *σ* are the Euclidean distances and the mean standard deviation, respectively, for the compounds in the training set. *Z* is an empirical cutoff value, which was set to 0.5 in this study.

### 2.3. Molecular Docking

Molecular docking was performed using the Molegro Virtual Docker v6.0.1 (MVD) software [116], and six targets were selected for anchorage studies (Table 2). The 3D structures of the enzymes used in this study were obtained from Protein Data Bank (PDB) [117], using the following codes: PDB ID 4Y46 for JNK-3; PDB ID 4Y14 for PTPB1; PDB ID 6FY4 for NQO1; PDB ID 3B2R for PDE5; PDB ID 5KIR for COX-2; and PDB ID 4NOS for *i*NOS. We did not dock the enzymes Nrf2 and NOX1 because 3D structures were not available in PDB for the human species. Initially, all water molecules were removed from the crystalline structure, and the root-mean-square deviation (RMSD) was calculated from the poses, which indicates the degree of reliability for the fit. The RMSD provides for the connection mode close to the experimental structure and is considered successful if the value is below 2.0 Å. The MolDock score algorithm was used as a scoring function, to predict the best interactions between the ligand and the receptor. Then, the anchor assistant was generated, in which the enzyme and ligands were inserted to analyze the stability of the system based on the interactions identified with the active site of the enzyme.

### 2.4. Prediction of ADMET Properties

ADME parameters were calculated using the SwissADME open-access web tool (http://www.swissadme.ch) [118], which offers a set of rapid predictive models for the assessment of physicochemical, pharmacokinetic, and pharmacological properties. The toxicity prediction was performed in OSIRIS Property Explorer (https://www.organic-chemistry.org/prog/peo/) [119], based on the following parameters: mutagenicity, tumorigenicity, reproductive effects, and irritability. For absorption, factors included membrane permeability, intestinal absorption, and substrate or inhibitor of P glycoprotein. Thus, we investigated compounds that did not exceed more than two violations of Lipinski's rule and for which the log*P* consensus was not greater than 4.15. In addition, compounds were not substrates for the permeability glycoprotein enzyme (P-gp). The distribution was assessed by factors that include the blood-brain barrier (logBB) and the permeability of the CNS. Metabolism was predicted based on the CYP substrate or inhibition models (CYP1A2, CYP2C19, CYP2C9, CYP2D6, and CYP3A4).

## 3. Results and Discussion

### 3.1. QSAR Modelling

The metrics mentioned are the most commonly used metrics for chemoinformatics, although others can be used to guarantee the high predictability of the model, such as ROC curves [120]. The results of the ROC curve and MCC analyses revealed excellent results. The models achieved ROC curves greater than 0.78 during cross-validation, and the MCC values were also greater than 0.52 during the cross-validation, revealing a model with excellent classification, performance, and robustness (Table 3, Figure S1). Only the model for the Nrf2 enzyme achieved an MCC below 0.5.  Table 4 shows the ROC curve values for each protein.

Using the models created, with excellent performance, the lignan set was screened to select compounds that are potentially active against the studied enzymes. Lignans with a probability of biological activity above 0.5 and that passed the applicability domain were considered active.

The results showed that no lignans were considered active for the JNK-3, PDE5, and COX-2 targets. However, 22 compounds were potentially active against the PTPB1 enzyme with a probability ranging from 50 to 74%, 111 compounds active against Nfr2 with a probability ranging from 50 to 64%, six compounds active against NOX1 with a probability ranging between 63 and 78%, and 27 compounds active against *i*NOS with probability varying between 52 and 79%.

### 3.2. Docking Molecular

The molecular docking study was performed for six enzymes that were targeted for the AD treatment. The lignan set was analyzed to select molecules with good probabilities for potential inactivation and activation activity against the enzymes targeted for AD treatment. Docking was not performed for Nrf2 and NOX1, due to the unavailability of human 3D protein structures.

In this study, the docking results were validated by the redocking of the crystallographic ligand and by the RMSD of the poses. Redocking consists of positioning and predicting the binding affinity of the crystallographic ligand in the region of the active site of the enzyme. The RMSD compares and calculates the mean deviation of the square root of the poses obtained by redocking and the structure of the ligand obtained experimentally. For the fit to be reliable, the RMSD value must be 2.0 Å or less. The results showed that the targets JNK-3, PTP1B, NQO1, PDE5, COX-2, and *i*NOS obtained RMSD values of 0.56, 0.25, 0.18, 0.47, 0.19, and 0.16 Å, respectively.

The Molegro software is capable of generating interaction energies for lignans, by producing a MoldockScore for each studied protein. Then, calculations were performed to identify the lignans with the best active potential probabilities for each analyzed protein, using the following formula:

(3) $$\begin{array}{c} \text{Prob}=\frac{E_{\text{Lig}}}{E_{\text{MLig}}},\text{se }E_{\text{Lig}}<E_{\text{Inib}}, \end{array}$$

where *E*_Lig_ is the energy of the analyzed lignan, *E*_MLig_ is the lowest energy obtained from the tested lignans, and *E*_Inib_ is the energy of the inhibitor ligand, obtained from the crystallography data for the tested protein. Only molecules that obtained binding energies below the binding energy for the crystallographic inhibitor ligand were considered to be potentially active.

Table 5 shows the interaction energies of the inhibitor ligand for each protein, and the top ten lignans with the best energy values for each protein.

Among the 159 lignans analyzed by molecular docking, 21 were found to be potentially active against JNK-3, 1 was identified for PTP1B, 157 were identified for NQO1, 34 were identified for PDE5, 53 were identified for COX-2, and 156 were identified for *i*NOS. These results indicated that lignans, in general, are more likely to activate the NQO1 and *i*NOS proteins and are not selective for the PTP1B enzyme.

### 3.3. Combined Analysis Based on Ligand and Structure

A second consensus analysis was performed to identify potential multitarget lignans, which demonstrate active potential probabilities for more than one protein, based on the RF model and docking. In this case, we use all the results of prediction of biological activity of the lignans and combine them with the results of docking. For this analysis, the following formula was used:

(4) $$\begin{array}{c} {\text{Prob}}_{\text{Comb}}=\frac{\left(\text{ProbDc}+\left(1+\text{ESP}\right)\times P_{\text{Activity}}\right.}{2+\text{ESP}},\text{Se }{\text{Prob}}_{\text{Comb}}>0.5, \end{array}$$

where Prob_Dc_ is the active potential probability from the molecular coupling analysis, ESP is the average specific value of the RF model, and *P*_Activity_ is the active potential probability value of the RF model. This combined probability was conditioned, as only molecules with values greater than 0.5 were considered likely to be active. Combined probability values were calculated for the lignans identified for each target enzyme, and we analyzed which molecules were multitarget.

After performing the combined analysis, based on the ligand and structure, and using the formula to identify multitarget molecules, we identified 139 molecules that were potentially active for two or five target enzymes, out of the entire lignan set analyzed. For Nrf2 and NOX1, we only used the biological activity probability data, and for NQO1, we only used the docking data not enough data was available for these enzymes to construct the necessary models.

The combined probability (Prob_Comb_), based on both ligand and structure, can increase the predictive power of the models and decrease the number of false positives. Combined probability analyses could be performed for five enzymes (JNK-3, PTP1B, PDE5, COX-2, and *i*NOS). For enzymes without sufficient data to build both models, only model was used. For molecules to be considered potentially active, the probability values should be equal to or greater than 0.5. However, for Prob_Dc_, the probability value should also be greater than that for the crystallographic ligand.

After the combined probability analysis, we selected the multitarget compounds that passed the applicability domain for all enzymes in this study. Using Prob_Comb_, we were able to select three compounds with probabilities of activity ranging from 50% to 61% for JNK-3, 43 compounds with a 52-72% probabilities for PTP1B, 57 compounds with 51%–72% probabilities for PDE5, 27 compounds with probabilities between 50% and 61% for COX-2, and 27 compounds with probabilities between 50% and 81% for *i*NOS (Table 6). The number of compounds with excellent combined probabilities was reduced when compared with the results of the docking probabilities; however, the combined probabilities increased the numbers of true positives.

Based on the biological activity probability data, 111 compounds, with probabilities ranging from 50% to 64%, were identified for Nrf2, and nine compounds, with probabilities ranging from 51% to 78%, were identified for NOX1. Based on the docking probability data, 156 compounds were selected, with probabilities ranging from 27% to 100%, for NQO1. For this enzyme, compounds with probabilities above 0.27 were considered, as these were greater than the probability of the crystallographic ligand, which was 0.26.

We observed that although the results of QSAR do not indicate active compounds for JNK-3, PDE5, and COX-2, after the application of the formula that combines prediction values of biological activity and docking (Prob_Comb_), we were able to identify active compounds for all targets of the study.

### 3.4. Prediction of ADMET Properties

The set of 139 potentially active and multitarget lignans were submitted to several predictive parameters to identify the compounds with the best ADMET profiles. Using physical-chemical properties, we attempted to verify compounds with good absorption, considering the lipid rule as a parameter.

According to Shimohama et al. [42, 43], molecules with molecular weights below 500 Da, calculated log*P* (Clog*P*) values less than five, less than five hydrogen bond donors, no more than ten hydrogen bond acceptors, and ≤10 rotating bonds have excellent absorption and bioavailability. Molecules that violate two or more of these rules do not show good absorption. We observed that 66% (92) of our lignans set showed solubility values that varied between soluble and moderately soluble.

Factors such as lipophilicity and solubility contribute to drug distribution *in vivo*, which is a requirement for advancing to preclinical and clinical tests. The most common descriptor for lipophilicity is the partition coefficient between *n*-octanol: water (log*P*). Ideal log*P* values are below 5.0. The results showed that 87% (121) of our lignan compounds had ideal log*P* values.

Metabolism can affect drug activity by changing the half-life, promoting the generation of toxic metabolites, or disrupting therapeutic potential. Pharmacokinetics are essential for understanding drug metabolism in the body. For a compound to display the desired effect during AD treatment, the drug must be able to cross the blood-brain barrier. Many compounds that have been developed fail at the preclinical and clinical testing stage due to metabolism effects and poor absorption in the brain. Currently, the prediction and selection of compounds that act on nervous system tissues can be performed through *in silico* tests. The results showed that among lignans that target three or more enzymes, nine lignans would likely cross the blood-brain barrier.

**Figure 1:**
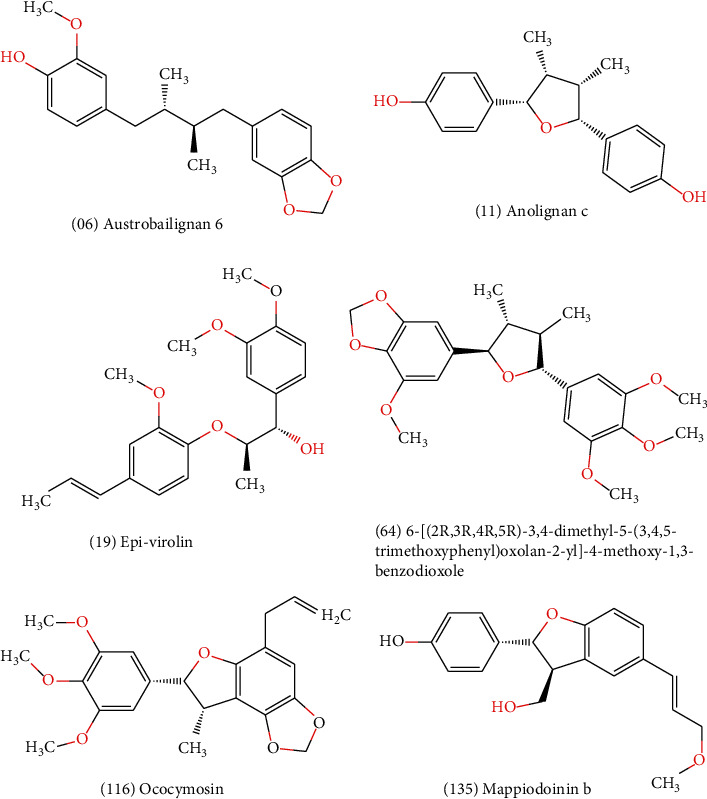
Lignans considered to be potentially active according to the Random Forest model, with multitarget effects and no predicted toxicity.

Toxicity was also evaluated, and among the compounds that appeared likely to cross the blood-brain barrier, compounds 6, 11, 19, 64, 116, and 135 had no predicted mutagenicity or tumorigenesis effects or negative effects on the reproductive system and irritability. Therefore, these molecules were considered to have the best ADMET properties because they do not present any toxicity risks. Tables S2 and S3 show the ADMET profiles of compounds with potential activity and multitargeting effects against four or more enzymes. In addition, Table S4 and Figure 1 show the compounds that did not present toxicity for these evaluated parameters.

Due to the antioxidant properties of lignans, the present study sought to perform a virtual screening among diverse structural lignans to identify potential molecules for the treatment of AD. Lignans represent a huge class of pharmacologically active compounds that exhibit various functionalities, which are worth exploring by pharmaceutical industries [121].

According to a review by Zálešák et al. [122], several researchers have identified the antioxidant activity and neuroprotective properties of lignans. Lignans isolated from *Schisandra bicolor* var. were assayed for their neuroprotective effects against SH-SY5Y cell damage induced by A*β*25−35. Among the active compounds, both new lignans (esquibitubina B (L1-4), F (L1-7), H (L4-1), and I) and previously isolated lignans (galgravine, (-)-nectandrin A, (-)-futocadsurine A, (+)-9′-hydroxigalbelgin, austrobailignan-6, oleiferin-F, (+)-dihydro-guaiaretic acid, and (-)- isootobafenol) increased the cell viability in SH-SY5Y cells, following the induction of cellular injury by 3.25 nM A*β*25-35 compared with the negative control group. Furthermore, 25 *μ*M dibenzocyclooctadiene lignans (L6-14 and NL5-10) from *Schisandra chinensis* exhibited protective activity against A*β*1-42 neurotoxicity induced in PC12 cells, increasing cell viability to 84.1% ± 5.4% and 82.1% ± 4.3%, respectively, compared with the control (52.0% ± 3.2%) [122].

Lignans are a large group of naturally occurring phenols widespread in the plant kingdom. In addition, notable advances have been made in the isolation and identification of lignans the last few years, which has already led to around 500 new congeners [121]. In addition, several studies have reported the synthesis of different lignans successfully and which have been tested for various biological activities.

### 3.5. Interaction Analysis

We analyzed the interactions of six lignans through molecular docking that obtained the highest probability of activity, multitarget, and with low toxicity. In addition, we consider analyzing the targets on which these compounds were most active.

**Figure 2:**
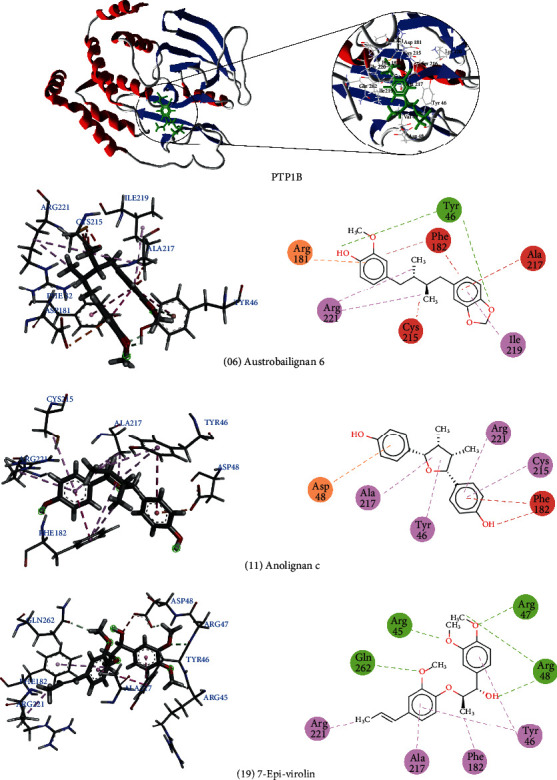
3D and 2D interactions between lignans and PTP1B. Hydrogen bonds are highlighted in green, hydrophobic interactions are highlighted in pink, steric interactions are highlighted in red, and electrostatic interactions are highlighted in orange.

The compounds austrobailignan 6 (06), anolignan c (11), and 7-Epi-virolin (19) formed several interactions with the PTP1B active site. Austrobailignan 6 formed hydrophobic interactions with residues Ile219 and Arg221, steric interactions with the amino acids Phe182, Cys215, and Ala217, an electrostatic bond with Arg181, and a hydrogen bond with Tyr46, stabilizing the bond. Analignan c formed four hydrophobic interactions with the amino acids Tyr26, Cys215, Ala217, and Arg221. In addition, it formed an electrostatic and a steric interaction. 7-Epi-viroline formed several hydrophobic interactions with Tyr46, Phe182, Ala217, and Arg221. Three important hydrogen bonds were also observed with the residues Arg47, Arg45, and Glu262 (Figure 2).

According to the study carried out by Krishnan et al. [123], the inhibitor CPT157633 managed to form electrostatic interactions with the PTP1B active site. In that study, interactions with the amino acids Cys215, Arg221, and Gln262 were reported. We observed that these amino acids are also interacting with lignans, forming more stable bonds.

**Figure 3:**
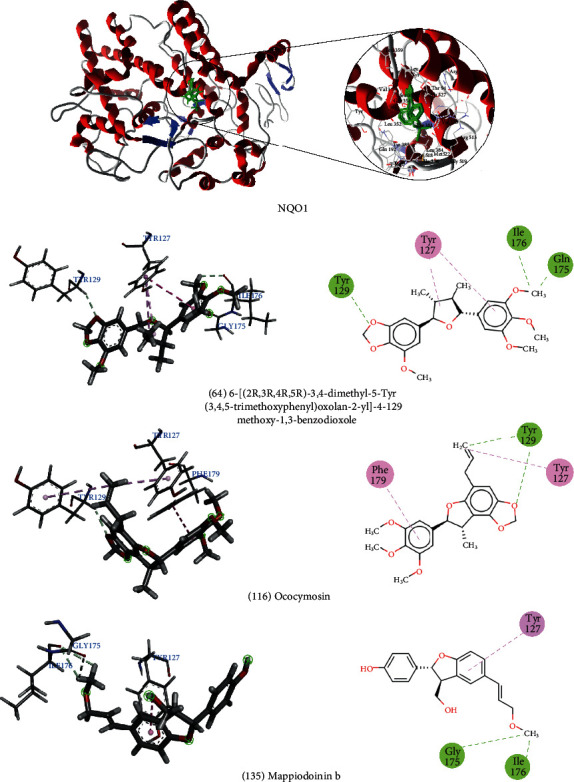
3D and 2D interactions between lignans and NQO1. Hydrogen bonds are highlighted in green, and hydrophobic interactions are highlighted in pink.

These same lignans were also investigated for their interactions with the NQO1 target. We found that 6 - [(2R, 3R, 4R, 5R) -3,4-dimethyl-5- (3,4,5-trimethoxyphenyl) oxolan-2-yl] -4-methoxy-1,3-benzodioxole (64) formed hydrogen bonds with the amino acids Tyr129, Gly175, and Ile176, and a hydrophobic interaction with the amino acid Tyr127. Oocymosin (116) showed hydrophobic interactions with Tyr127 and Phe179. In addition, it formed a hydrogen bond with the Tyr129 residue. Mappiodionin b (135) formed hydrogen bonds with Gly175 and Ile176 and a hydrophobic interaction with Tyr127. All compounds formed interactions with the same amino acids (Figure 3).

NQO1 must be activated to display antioxidant activity. According to Strandback et al. [124], the addition of N-(2-bromophenyl)pyrrolidine-1-sulfonamide (BPPSA) stabilized the flexible C-terminal region of the protein, resulting in the slower incorporation of deuterium. The amino acids involved in the bond were Tyr127, Thr128, and the catalytic residues Tyr156 and His162.

**Figure 4:**
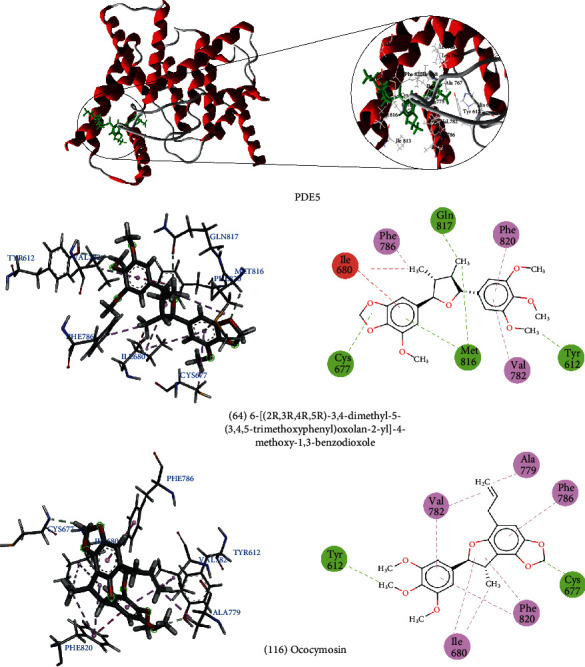
3D and 2D interactions between lignans and PDE5. Hydrogen bonds are highlighted in green, hydrophobic interactions are highlighted in pink, and steric interactions are highlighted in red.

Compounds 6-[(2*R*,3*R*,4*R*,5*R*)-3,4-dimethyl-5-(3,4,5-trimethoxyphenyl)oxolan-2-yl] -4-methoxy-1, 3-benzodioxole (64) and Ococymosin (116) interacted well with PDE5. Compound 64 was able to form three hydrogen bonds with Met816, Tyr612, and Gln817 and four hydrophobic interactions with the amino acids Cys677, Val782, Phe786, and Phe820. It also formed a steric interaction with Ile680. Ococymosin formed two hydrogen bonds with Tyr612 and Cys677 and five hydrophobic interactions with Ile680, Ala779, Val782, Phe786, and Phe820 (Figure 4).

Experimental studies carried out by Wang et al. [125] showed that the drug vardenafil is a potent PDE5 inhibitor, binding to several amino acids in the active site. The amino acids that interacted with vardenafil are Tyr612, Leu765, Ile768, Ala767, Ile680, Cys677, Ty676, Ile813, Met816, Gln817, and Phe820. Most of these amino acids also interacted with lignans.

## 4. Conclusions

AD is a complex and multifactorial disease, comprising a variety of aberrant cellular and molecular processes in different cell types and brain regions. The activation and inactivation of a variety of enzymes can contribute to neuroprotection or disease progression. Therefore, AD therapy must be able to block or compensate for various abnormal pathological events [38].

Few drugs are available for AD treatment. In addition, AD pathophysiology is not well-understood, and the identification of targets for disease treatment remains a major challenge for drug discovery. Therefore, in this study, we investigated several potential targets that are directly and indirectly involved in the development and progression of AD, through oxidative stress mechanisms, aiming to explore new targets and to design effective drugs, with minimal side effects, for AD treatment. We examined a set of lignans and used virtual screening to select compounds with potential multitargeting effects for the treatment of AD.

The predictive models built in this study obtained excellent performance results, with accuracies greater than 73%. To increase the predictive power and decrease the number of false positives generated by these models, a combined analysis was used, based on both ligand and structure. The combined analysis was able to identify potentially active molecules, based on the Random Forest and multitargeting models.

Out of 159 total lignans, several potentially active compounds were identified: three compounds with probabilities of activity ranging from 50% to 61% for JNK-3, 43 compounds with a 52-72% probabilities for PTP1B, 57 compounds with 51%–72% probabilities for PDE5, 27 compounds with probabilities between 50% and 61% for COX-2, and 27 compounds with probabilities between 50% and 81% for *i*NOS; 111 compounds with probabilities ranging from 50% to 64% were identified for Nrf2; nine compounds with probabilities ranging from 51% to 78% were identified for NOX1, and 156 compounds were selected, with probabilities ranging from 27% to 100%, for NQO1. We also identified 139 potentially active molecules for two to five target enzymes, from the entire lignan set analyzed.

Among the 139 lignans that were considered to be potentially active and multitargeting, 92 showed good absorption, bioavailability, and solubility, ranging from soluble to moderately soluble. Among the compounds that were considered to be multitargeting, we selected those likely to cross the blood-brain barrier, through an *in silico* evaluation, resulting in the identification of nine lignans, which were then evaluated for toxicity. The compounds austrobailignan (06), anolignan c (11), 7-epi-virolin (19), 6-[(2*R*,3*R*,4*R*,5*R*)-3,4-dimethyl-5-(3, 4,5-trimethoxyphenyl)oxolan-2-yl]-4-methoxy-1, 3-benzodioxole (64), ococymosin (116), and mappiodoinin b (135) were considered to have no toxicity risks for the evaluated parameters.

We suggest that lignans, especially austrobailignan (06), anolignan c (11), 7-epi-virolin (19), 6-[(2*R*,3*R*,4*R*,5*R*)-3,4-dimethyl-5-(3,4,5-trimethoxyphenyl)oxolan-2-yl]-4-methoxy-1, 3-benzodioxole (64), ococymosin (116), and mappiodoinin b (135), have high probability of activity against several enzymes that may be involved in AD pathogenesis and may confer neuroprotective effects, with low toxicity. The proposed compounds are projected as possible solutions that need to be validated experimentally.

## Figures and Tables

**Table 1:** Set of molecules from the ChEMBL databases for each enzyme selected in the study.

Database	Active molecules	Inactive molecules	Total
JNK-3	580 (pIC_50_ ≥ 6.0)	642 (pIC_50_ < 6.0)	1.222
PTP1B	1.446 (pIC_50_ ≥ 5.0)	1.354 (pIC_50_ < 5.0)	2.800
NFR2	163 (activity)	85 (no activity)	248
NOX1	85 (pIC_50_ ≥ 4.75)	60 (pIC_50_ < 4.75)	145
PDE5	873 (pIC_50_ ≥ 7.0)	869 (pIC_50_ < 7.0)	1742
COX2	2.018 (pIC_50_ ≥ 5.50)	1.702 (pIC_50_ < 5.50)	3.720
*i*NOS	396 (pIC_50_ ≥ 5.50)	367 (pIC_50_ < 5.50)	763

**Table 2:** Information regarding the selected enzymes, obtained from the PDB database and used for docking.

PDB ID	Enzyme	Class	PDB ligand	Resolution
4Y46	c-Jun N-terminal kinase	Transferase	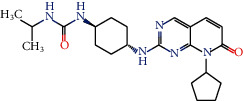	2.04 Å
4Y14	Tyrosine phosphatase 1B	Hydrolase	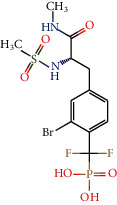	1.89 Å
6FY4	NAD(P)H:quinone oxidoreductase	Oxidoreductase	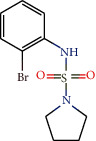	2.76 Å
3B2R	Phosphodiesterase-5	Hydrolase	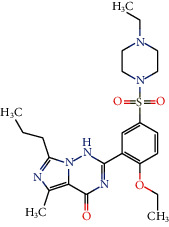	2.07 Å
5KIR	Cyclooxygenase-2	Oxidoreductase	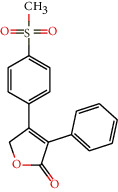	2.69 Å
4NOS	Inducible nitric oxide synthase	Oxidoreductase	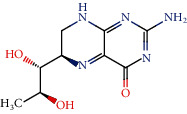	2.25 Å

**Table 3:** Performance summary corresponding with the results obtained for all Random Forest models.

Enzyme	Validation	Accuracy	Sensitivity	Specificity	PPV	NPV	MCC
JNK-3	Test	0.89	0.91	0.87	0.86	0.91	0.78
	Cross	0.83	0.85	0.82	0.81	0.85	0.67
PTP1B	Test	0.81	0.81	0.81	0.82	0.80	0.62
	Cross	0.82	0.82	0.82	0.83	0.81	0.64
NFR2	Test	0.76	0.75	0.76	0.86	0.61	0.50
	Cross	0.73	0.78	0.63	0.80	0.60	0.41
NOX1	Test	0.82	0.76	0.91	0.92	0.73	0.67
	Cross	0.80	0.89	0.66	0.92	0.73	0.58
PDE5	Test	0.87	0.9	0.84	0.85	0.9	0.75
	Cross	0.86	0.88	0.85	0.85	0.87	0.73
COX2	Test	0.78	0.83	0.71	0.77	0.78	0.55
	Cross	0.76	0.81	0.7	0.76	0.76	0.52
*i*NOS	Test	0.81	0.87	0.74	0.78	0.84	0.62
	Cross	0.8	0.85	0.74	0.78	0.82	0.60

**Table 4:** Values for the ROC curves, during the test and cross-validation, for each RF model.

Enzyme	ROC curve	
	Test	Cross
JNK-3	0.96	0.91
PTP1B	0.87	0.89
NFR2	0.82	0.81
NOX1	0.90	0.78
PDE5	0.95	0.94
COX2	0.84	0.84
*i*NOS	0.87	0.87

**Table 5:** MoldockScore scores for the top ten lignans with the best energy values relative to the energy value of the crystallographic ligand for each protein.

ID	JNK-3	PTP1B	NQO1	PDE5	COX2	*i*NOS
1	-183	-177	-137	-204	-203	-178
2	-175	-156	-137	-192	-193	-153
3	-164	-154	-136	-182	-191	-147
4	-159	-153	-124	-169	-190	-144
5	-155	-153	-120	-167	-187	-143
6	-148	-152	-116	-166	-176	-143
7	-148	-152	-116	-164	-175	-143
8	-146	-151	-114	-164	-174	-141
9	-146	-151	-112	-164	-172	-139
10	-144	-150	-108	-162	-170	-139
Ligand PDB	-134	-156	-36	-139	-142	-59

**Table 6:** Potentially active lignans, multitarget for four or more enzymes, based on the RF and docking model. In bold are the active enzymes that walk in the applicability domain.

ID	Prob_Comb_					Prob_Activity_		Prob_Dc_	Multitarget
	JNK-3	PTP1B	PDE5	COX-2	*i*NOS	NFR2	NOX1	NQO1	
05	0.39	**0.68**	**0.52**	0.41	**0.62**	**0.54**	0.17	**0.47**	5
06	0.38	**0.67**	0.49	0.43	0.59	**0.57**	**0.51**	**0.35**	4
07	0.45	**0.66**	**0.56**	0.53	0.70	**0.59**	0.25	**0.49**	4
11	0.35	**0.64**	0.48	0.46	0.53	**0.53**	**0.63**	**0.38**	4
12	0.37	**0.62**	**0.51**	0.48	0.60	**0.60**	0.25	**0.64**	4
13	0.32	**0.62**	**0.59**	0.46	0.57	**0.51**	0.45	**0.72**	4
14	0.51	**0.62**	**0.54**	0.45	**0.62**	**0.60**	0.25	**0.45**	5
19	0.31	**0.59**	0.48	0.49	0.56	**0.56**	**0.51**	**0.35**	4
33	0.41	**0.53**	**0.51**	**0.50**	0.51	**0.58**	0.41	**0.39**	5
34	0.35	**0.53**	**0.50**	0.43	**0.54**	**0.56**	0.45	**0.40**	5
35	0.27	**0.52**	**0.59**	0.46	0.49	**0.61**	**0.70**	**0.56**	5
38	0.54	**0.52**	**0.69**	**0.51**	0.66	**0.52**	0.31	**1.00**	5
39	0.54	**0.52**	0.68	**0.61**	0.65	**0.61**	0.33	**0.81**	4
41	0.59	**0.52**	0.61	**0.60**	0.54	**0.58**	0.33	**0.67**	4
42	0.52	**0.52**	0.64	**0.59**	0.62	**0.57**	0.36	**0.78**	4
44	0.35	**0.51**	**0.52**	0.45	0.58	**0.54**	0.43	**0.50**	4
45	0.45	**0.51**	**0.52**	0.49	0.55	**0.54**	0.35	**0.47**	4
47	0.39	**0.50**	**0.56**	0.47	0.54	**0.56**	0.36	**0.56**	4
52	0.45	0.48	0.49	**0.50**	**0.69**	**0.54**	0.45	**0.38**	4
104	0.42	0.40	**0.55**	**0.51**	0.31	**0.59**	0.30	**0.50**	4
106	0.48	0.40	**0.58**	**0.57**	0.52	**0.56**	0.25	**0.63**	4
108	**0.53**	0.40	**0.56**	0.45	0.64	**0.50**	0.38	**0.66**	4
115	0.31	0.39	**0.52**	**0.51**	0.61	**0.52**	0.28	**0.52**	4
134	0.26	0.36	**0.50**	0.40	**0.51**	**0.56**	**0.71**	**0.38**	5
141	0.40	0.35	**0.54**	**0.50**	0.50	**0.62**	**0.52**	**0.40**	5
142	0.37	0.35	**0.50**	**0.51**	0.65	**0.55**	0.30	**0.40**	4
146	0.40	0.33	**0.57**	**0.56**	0.66	**0.55**	0.27	**0.60**	4
153	0.49	0.32	**0.60**	**0.50**	0.63	**0.52**	0.39	**0.74**	4

## Data Availability

The data used to support the findings of this study are available from the corresponding author upon request.

## Supplementary Materials

Supplementary Materials Table S1: smiles of the lignans used in the study. Figure S1: ROC curves generated from the RF models, for each studied enzyme. Table S2: the physical and chemical properties of lignans that are considered to be potentially active against Alzheimer's disease and have multitargeting effects against four or more enzymes. Table S3: pharmacokinetic properties of the lignans that are considered to be potentially active against Alzheimer's disease and have multitargeting effects against four or more enzymes. Lignans that are predicted to cross the blood-brain barrier are highlighted in bold. Table S4: toxicity evaluations for lignans with the best ADMET profiles that are potentially active and that have multitarget effects against four or more enzymes. Lignans that did not present toxicity for any of the analyzed parameters are highlighted in bold.
