# Supplementary material for: Identification of New Targets and the Virtual Screening of Lignans against Alzheimer's Disease
Source: Oxid Med Cell Longev. 2020 Aug 15;2020:3098673. doi: 10.1155/2020/3098673 (PMC7448245; doi:10.1155/2020/3098673)
Supplement: Supplementary Materials — Table S1: smiles of the lignans used in the study. Figure S1: ROC curves generated from the RF models, for each studied enzyme. Table S2: the physical and chemical properties of lignans that are considered to be potentially active against Alzheimer's disease and have multitargeting effects against four or more enzymes. Table S3: pharmacokinetic properties of the lignans that are considered to be potentially active against Alzheimer's disease and have multitargeting effects against four or more enzymes. Lignans that are predicted to cross the blood-brain barrier are highlighted in bold. Table S4: toxicity evaluations for lignans with the best ADMET profiles that are potentially active and that have multitarget effects against four or more enzymes. Lignans that did not present toxicity for any of the analyzed parameters are highlighted in bold. [file 3098673.f1.docx]

**Identification of new targets and the virtual screening of lignans against Alzheimer's disease**

Mayara dos Santos Maia, ^1^ Gabriela Cristina Soares Rodrigues, ^1^ Natália Ferreira de Sousa, ^1^ Marcus Tullius Scotti, ^1^ Luciana Scotti, ^1^* and Francisco Jaime B. Mendonça-Junior ^2^

^1^Laboratory of Cheminformatics, Program of Natural and Synthetic Bioactive Products (PgPNSB), Health Sciences Center, Federal University of Paraíba, João Pessoa-PB, Brazil
^2^Laboratory of Synthesis and Drug Delivery, State University of Paraíba, João Pessoa-PB, Brazil .

*Correspondence should be addressed to Luciana Scotti; luciana.scotti@gmail.com

Table S1: Smiles of the lignans used in the study.

| **ID** | **SMILES** |
| --- | --- |
| 1 | O[c]([cH][cH]1)[c](O)[cH][c]1C2OC(=O)C3C2COC3[c]4[cH][c](O)[c](O)[cH][cH]4 |
| 2 | OCC(O)C[c]([cH]1)[cH][cH][c](O)[c]1[c]([cH]2)[cH][cH][c](O)[c]2CC=C |
| 3 | O[c]([c]21)[c](OC)[c](OC)[cH][c]2CC(C)C(C)C1[c]([cH]3)[cH][c]4OCO[c]4[c]3OC |
| 4 | OC(C(C)C(C)C1)[c]2[cH][c]3OCO[c]3[c](OC)[c]2[c]4[c]1[cH][c](OC)[c](OC)[c]4OC(=O)C(C)=CC |
| 5 | O=C(C(C)CC)O[c]1[c]2[c]3[c](OC)[c]4OCO[c]4[cH][c]3C(O)C(C)C(C)C[c]2[cH][c](OC)[c]1OC |
| 6 | O[c]([cH][cH]1)[c](OC)[cH][c]1CC(C)C(C)C[c]2[cH][c]3OCO[c]3[cH][cH]2 |
| 7 | O=C(C21)OCC2C[c]3[cH][c]4OCO[c]4[cH][c]3C1[c]([cH]5)[cH][c]6OCO[c]6[c]5OC |
| 8 | O[c]([cH][cH]1)[cH][c](O)[c]1CC(=C)C(=C)C[c]2[cH][c]3OCO[c]3[cH][cH]2 |
| 9 | O[c]([cH][cH]1)[cH][cH][c]1CC(=C)C(=C)C[c]2[cH][cH][c](O)[cH][cH]2 |
| 10 | O[c]([cH][cH]1)[cH][cH][c]1C(O2)C(C)C(C)C2[c]3[cH][cH][c](O)[cH][cH]3 |
| 11 | O=C(C21)OCC2C[c]3[cH][c]4OCO[c]4[cH][c]3C1[c]([cH]5)[cH][c]6OCO[c]6[c]5OC |
| 12 | O=C1OCC2C1COC2[c]([cH]3)[cH][c](OC)[c](OC)[c]3OC |
| 13 | OC(CO1)C(O)C(O)C1O[c]([c]2COC([c]32)=O)[c]4[cH][c](OC)[c](OC)[cH][c]4[c]3[c]5[cH][c]6OCO[c]6[cH][cH]5 |
| 14 | O=C1OCC(C[c]2[cH][c]([c](O)[cH][cH]2)OC)C1=C[c]([cH]3)[cH][cH][c](O)[c]3OC |
| 15 | O[c]([cH][cH]1)[cH][cH][c]1CC(=C)C(=C)C[c]2[cH][cH][c](OC)[cH][c]2O |
| 16 | OC(C[c]1[cH][cH][c]([cH][c]1(O))OC)(CO)C(=C)C[c]2[cH][cH][c](O)[cH][cH]2 |
| 17 | OC([c]1[cH][cH][c]([c](OC)[cH]1)OC)C(C)O[c]2[c](OC)[cH][c](CC=C)[cH][c]2OC |
| 18 | OC([c]1[cH][cH][c]([c](OC)[cH]1)OC)C(C)O[c]2[cH][cH][c](C=CC)[cH][c]2OC |
| 19 | OCC1C([c]2[cH][cH][c]([c]([cH]2)OC)O)OCC1C[c]([cH]3)[cH][cH][c](O)[c]3OC |
| 20 | O[c]1[cH][c]2COC[c]2[cH][c]1CC(C)C(C)C[c]3[cH][c]4OCO[c]4[cH][c]3O |
| 21 | O=C(C=C1OC)C(CC=C)=CC1(OC)C(C)=C[c]([cH]2)[cH][cH][c](O)[c]2OC |
| 22 | O=C1C=C(OC)C(OC)=CC1(CC=C)C(C)C(OC(C)=O)[c]([cH]2)[cH][cH][c](OC)[c]2OC |
| 23 | O=C(C(C)C1(C))[c]2[cH][c](OC)[c](OC)[cH][c]2C1[c]3[cH][c]4OCO[c]4[cH][cH]3 |
| 24 | O=C(C(C)C1(C))[c]2[cH][c](OC)[c](OC)[cH][c]2C1[c]([cH]3)[cH][cH][c](OC)[c]3OC |
| 25 | O=C(C(C)C1(C))[c]2[cH][c](OC)[c](OC)[cH][c]2C1[c]([cH]3)[cH][cH][c](OC)[c]3OC |
| 26 | O=C(C(C)C1(C))[c]2[cH][c](OC)[c](OC)[cH][c]2C1[c]([cH]3)[cH][cH][c](OC)[c]3OC |
| 27 | O=C(C(C)C1(C))[c]2[cH][c]3OCO[c]3[cH][c]2C1[c]([cH]4)[cH][cH][c](OC)[c]4OC |
| 28 | O=C(C(C)C1(C))[c]2[cH][c](OC)[c](OC)[cH][c]2C1[c]3[cH][c]4OCO[c]4[cH][cH]3 |
| 29 | OCC1C([c]2[cH][cH][c]([cH][cH]2)O)OCC1C(OC)[c]3[cH][cH][c](O)[cH][cH]3 |
| 30 | OC1OCC(C[c]2[cH][c]([c](O)[cH][cH]2)OC)C1C[c]([cH]3)[cH][cH][c](O)[c]3OC |
| 31 | OC1OCC(C[c]2[cH][c]([c](O)[cH][cH]2)OC)C1C[c]([cH]3)[cH][cH][c](O)[c]3OC |
| 32 | O[c]([cH][cH]1)[c](OC)[cH][c]1CC2COC(OCCCC)C2C[c]([cH]3)[cH][cH][c](O)[c]3OC |
| 33 | O[c]([cH][cH]1)[c](OC)[cH][c]1CC2COC(OCC)C2C[c]([cH]3)[cH][cH][c](O)[c]3OC |
| 34 | CO[c]1[cH][c](CC=C)[cH][c]([cH]2)[c]1o[c]2[c]3[cH][c]4OCO[c]4[cH][cH]3 |
| 35 | OCC(C([c]1[cH][cH][c]([c]([cH]1)OC)O)OCC)O[c]([c]([cH]2)OC)[c](OC)[cH][c]2C(C3)O[c]4[cH][c](O)[cH][c](O)[c]4C3=O |
| 36 | O=C([c]1[cH][cH][c]([cH][cH]1)O)OCC(C([c]2[cH][cH][c]([c]([cH]2)OC)O)OCC)O[c]([c]([cH]3)OC)[c](OC)[cH][c]3C(C4)O[c]5[cH][c](O)[cH][c](O)[c]5C4=O |
| 37 | OCC(C([c]1[cH][cH][c]([c]([cH]1)OC)O)OCC)O[c]([c]([cH]2)OC)[c](OC)[cH][c]2[c]([cH]3)o[c]4[cH][c](O)[cH][c](O)[c]4c3=O |
| 38 | O=C([c]1[cH][cH][c]([c](O)[c]1(OC))OC)C(COC([c]2[cH][cH][c]([cH][cH]2)O)=O)O[c]([c]([cH]3)OC)[c](OC)[cH][c]3C=CCOC(=O)[c]4[cH][cH][c](O)[cH][cH]4 |
| 39 | O=C([c]1[cH][cH][c]([c](O)[c]1(OC))OC)C(COC([c]2[cH][cH][c]([cH][cH]2)O)=O)O[c]([c]([cH]3)OC)[c](OC)[cH][c]3C(O)C(O)COC(=O)[c]4[cH][cH][c](O)[cH][cH]4 |
| 40 | OC([c]1[cH][cH][c]([c](O)[c]1(OC))OC)C(COC([c]2[cH][cH][c]([cH][cH]2)O)=O)O[c]([c]([cH]3)OC)[c](OC)[cH][c]3C=CCOC(=O)[c]4[cH][cH][c](O)[cH][cH]4 |
| 41 | OC([c]1[cH][cH][c]([c](O)[c]1(OC))OC)C(COC([c]2[cH][cH][c]([cH][cH]2)O)=O)O[c]([cH][cH]3)[c](OC)[cH][c]3C=CCOC(=O)[c]4[cH][cH][c](O)[cH][cH]4 |
| 42 | O[c]([cH]1)[c](OC)[cH][cH][c]1C(O)C(COC([c]2[cH][cH][c]([cH][cH]2)O)=O)O[c]([c]([cH]3)OC)[c](OC)[cH][c]3C=CCOC(=O)[c]4[cH][cH][c](O)[cH][cH]4 |
| 43 | O[c]([c](OC)[cH]1)[c](OC)[cH][c]1C(O)C(CO)O[c]2[c](OC)[cH][c](CC=C)[cH][c]2OC |
| 44 | OCC(CO)O[c]([cH][cH]1)[c](OC)[cH][c]1C(O)C(CO)O[c]([c]([cH]2)OC)[c](OC)[cH][c]2CCCO |
| 45 | OCCC[c]([cH][c]1OC)[cH][c](OC)[c]1OC(CO)C(O)[c]([cH][c]2OC)[cH][c](OC)[c]2OC(CO)CO |
| 46 | OCC(CO)O[c]([cH][cH]1)[c](OC)[cH][c]1C(O)C(CO)O[c]([c]([cH]2)OC)[c](OC)[cH][c]2CCCO |
| 47 | OCCC[c]([cH][c]1OC)[cH][c](OC)[c]1OC(CO)C(O)[c]([cH][c]2OC)[cH][c](OC)[c]2OC(CO)CO |
| 48 | OCC(CO)O[c]([cH][cH]1)[c](OC)[cH][c]1C(O)C(CO)O[c]([c]([cH]2)OC)[c](OC)[cH][c]2CCCO |
| 49 | O[c]([c]([c]1(OC))OC)[cH][c](CC(C2C)C)[c]1[c]3[c](OC)[c](OC)[c](O)[cH][c]3C2OC[c]4[cH][cH][cH][cH][cH]4 |
| 50 | O[c]([cH][cH]1)[c](OC)[cH][c]1C(O)C(CO)O[c]([c]([cH]2)OC)[c](OC)[cH][c]2[c]([cH]3)o[c]4[cH][c](O)[cH][c](O)[c]4c3=O |
| 51 | OC([c]2[cH][cH][c]1OCO[c]1([cH]2))C3COC(=O)C3C(=O)[c]4[cH][c]5OCO[c]5[cH][cH]4 |
| 52 | OC([c]2[cH][cH][c]1OCO[c]1([cH]2))C3COC(=O)C3C(=O)[c]4[cH][c]5OCO[c]5[cH][cH]4 |
| 53 | O=C(C)OC([c]1[cH][cH][c]([c](OC)[cH]1)OC)C2COC(=O)C2C(=O)[c]3[cH][c]4OCO[c]4[cH][cH]3 |
| 54 | O=C(C)OC([c]2[cH][cH][c]1OCO[c]1([cH]2))C3C(=O)OCC3C(=O)[c]4[cH][c]5OCO[c]5[cH][cH]4 |
| 55 | O[c]([cH][cH]1)[c](OC)[cH][c]1C(O)C(CO)O[c]([cH][cH]2)[c](OC)[cH][c]2C3OCC4C3COC4[c]([cH]5)[cH][c](OC)[c](O)[c]5OC |
| 56 | O[c]([cH][cH]1)[c](OC)[cH][c]1C(O)C(CO)O[c]([cH][cH]2)[c](OC)[cH][c]2CCCO |
| 57 | OCC(C[c]1[cH][c]([c]([c]([cH]1)OC)O)OC)OC(CC)C(OC)[c]([cH]2)[cH][cH][c](O)[c]2OC |
| 58 | OCC(C[c]1[cH][cH][c]([c]([cH]1)OC)O)OC(CC)C(OC)[c]([cH]2)[cH][c](OC)[c](O)[c]2OC |
| 59 | O[c]([cH][cH]1)[c](OC)[cH][c]1C(O)C(CO)O[c]([cH][cH]2)[c](OC)[cH][c]2CCCO |
| 60 | O[c]([c](OC)[cH]1)[c](OC)[cH][c]1C2OCC3C2COC3[c]([cH]4)[cH][c](OC)[c](OC)[c]4OC |
| 61 | O=C1OCC2C1C([c]3[cH][c]([c](OC)[c](OC)[cH]3)OC)[c]4[cH][c]5OCO[c]5[cH][c]4C2OC(O6)C(O)C(O)C(O)C6COC(=O)C |
| 62 | O[c]([c](OC)[cH]1)[c](OC)[cH][c]1C(C2C3COC2(=O))[c]4[cH][c]5OCO[c]5[cH][c]4C3OC(O6)C(O)C(O)C(O)C6COC(O7)C(O)C(O)C(O)C7CO |
| 63 | CO[c]([cH]1)[c](OC)[c](OC)[cH][c]1C(O2)C(C)C(C)C2[c]([cH]3)[cH][c]4OCO[c]4[c]3OC |
| 64 | CO[c]([cH]1)[c]2OCO[c]2[cH][c]1C(O3)C(C)C(C)C3[c]([cH]4)[cH][c]5OCO[c]5[c]4OC |
| 65 | O=C(C)OC([c]1[cH][cH][c]([c](OC)[cH]1)OC)C(C)O[c]2[c](OC)[cH][c](CC=C)[cH][c]2OC |
| 66 | O=C(C21)OCC2C(O)[c]3[c](OC)[c]4OCO[c]4[cH][c]3C1[c]5[cH][c]6OCO[c]6[cH][cH]5 |
| 67 | OCC1C2C(=O)OC1[c]3[c](OC)[c]4OCO[c]4[cH][c]3C2[c]5[cH][c]6OCO[c]6[cH][cH]5 |
| 68 | O[c]([cH][cH]1)[c]([c]2[cH][c]([cH][cH][c]2(O))CC=C)[cH][c]1C(O3)C(C)OC(C)C3[c]([cH]4)[cH][cH][c](O)[c]4[c]([cH]5)[c](O)[cH][cH][c]5CC=C |
| 69 | OC(C)C(O)[c]1[cH][c]2[c]3[cH][c](CC=C)[cH][cH][c]3O[c]4[cH][cH][c](C(C(O)C)O)[cH][c]4[c]5[cH][c](CC=C)[cH][cH][c]5O[c]2[cH][cH]1 |
| 70 | O[c]1[cH][c](CC=C)[cH][cH][c]1O[c]([cH][cH]2)[cH][cH][c]2C=CC=O |
| 71 | OC(C)C(O)[c]([cH]1)[cH][cH][c](O)[c]1[c]([cH]2)[c](O)[cH][cH][c]2CC=C |
| 72 | OCC(O)C[c]([cH]1)[cH][cH][c](O)[c]1[c]([cH]2)[c](O)[cH][cH][c]2CC=C |
| 73 | OCC(O)C[c]([cH]1)[cH][cH][c](O)[c]1[c]2[cH][c](CC=C)[cH][cH][c]2OC(O3)C(O)C(O)C(O)C3CO |
| 74 | O=C1OCC2C(C)[c]([c]43)[cH][cH][cH][c]4[cH][cH][cH][c]3C5C12[c]6[c]5[cH][c](OC)[c](OC)[c]6OC |
| 75 | O=C(O)[c]1[c]2CC(C)C(C)C(O)[c]3[cH][c]4OCO[c]4[c](OC)[c]3[c]2[c](OC)[c](OC)[c]1OC |
| 76 | O[c]1[c](C(O)=O)[c]2CC(C)C(C)C(O)[c]3[cH][c]4OCO[c]4[c](OC)[c]3[c]2[c](OC)[c]1OC |
| 77 | O=C(C)C[c]1[cH][c]2OCO[c]2[c](OC)[c]1[c]3[c](OC)[c]4OCO[c]4[cH][c]3CC(=O)C |
| 78 | O=C(C)C[c]1[cH][c](OC)[c](OC)[c](OC)[c]1[c]2[c](OC)[c](OC)[c](OC)[cH][c]2CC(=O)C |
| 79 | CO[c]1[cH][c]2CC(C)C(C)C(OCC(C)C)[c]3[cH][c](OC)[c](OC)[c](OC)[c]3[c]2[c](OC)[c]1OC |
| 80 | O[c]([cH][c]21)[c](OC)[c](OC)[c]2[c]3[c](OC)[c](OC)[c](OC)[cH][c]3CC(C)C(C)C1OCC(C)C |
| 81 | O[c]1[cH][c]2CC(C)C(C)C(OC(C(C)C)=O)[c]3[cH][c]4OCO[c]4[c](OC)[c]3[c]2[c](OC)[c]1OC |
| 82 | OC(OC)C=C[c]([cH]1)[cH][c](OC)[c](O2)[c]1C(CO)C2[c]([cH]3)[cH][cH][c](O)[c]3OC |
| 83 | OCC1[c]2[cH][c](C=CC(OC)OC)[cH][c](OC)[c]2OC1[c]([cH]3)[cH][cH][c](O)[c]3OC |
| 84 | O[c]([cH][cH]1)[c](OC)[cH][c]1C(O)C(CO)O[c]([cH][cH]2)[c](OC)[cH][c]2C3OCC4C3COC4[c]([cH]5)[cH][c](OC)[c](O)[c]5OC |
| 85 | OCC1O[c]2[cH][c](C=CC(O)=O)[cH][cH][c]2OC1[c]3[cH][c](O)[c](O)[cH][cH]3 |
| 86 | O[c]([cH][cH]1)[c](OC)[cH][c]1C(O2)C(CO)O[c]([cH]3)[c]2[cH][cH][c]3CCCO |
| 87 | O[c]([cH][cH]1)[c](OC)[cH][c]1C2O[c]3[cH][cH][c](C(O)=O)[cH][c]3OC2CO |
| 88 | OCC1O[c]2[cH][c](C=O)[cH][cH][c]2OC1[c]3[cH][c](O)[c](O)[cH][cH]3 |
| 89 | O[c]([cH][cH]1)[c](O)[cH][c]1C2OC(=O)C3C2COC3[c]4[cH][c](O)[c](O)[cH][cH]4 |
| 90 | OC([c]1[cH][cH][c]([c]([cH]1)OC)O)C2COC(OC)C2C[c]([cH]3)[cH][cH][c](O)[c]3OC |
| 91 | O[c]1[cH][c]2OCO[c]2[cH][c]1CC(C)C(C)C[c]([cH]3)[c](O)[cH][c](O)[c]3OC |
| 92 | O[c]1[cH][c](OC)[c](OC)[cH][c]1CC(C)C(C)C[c]2[cH][c]3OCO[c]3[cH][c]2O |
| 93 | O[c]1[cH][c]2OCO[c]2[cH][c]1CC(C)C(C)C[c]3[cH][c](O)[c](OC)[cH][c]3OC |
| 94 | O=C1C=C(OC)C(=O)C=C1CC(C)C(C)C[c]2[cH][c]3OCO[c]3[cH][c]2O |
| 95 | O[c]([cH][cH]1)[c](OC)[cH][c]1C2C3C[c]4[cH][c](O)[c](OC)[cH][c]4CC3CO2 |
| 96 | FCC(C(C)C)C([c]1[cH][c]([c](O)[cH][cH]1)OC)[c]([cH]2)[cH][cH][c](O)[c]2OC |
| 97 | O[c]([cH][cH]1)[c](OC)[cH][c]1C(C(C)C(C)COC)[c]([cH]2)[cH][cH][c](O)[c]2OC |
| 98 | O[c]([cH][cH]1)[c](OC)[cH][c]1C(C(C)C(C)COC)[c]([cH]2)[cH][cH][c](O)[c]2OC |
| 99 | O[c]([cH][cH]1)[c](OC)[cH][c]1C(C(C(C)C)COC)[c]([cH]2)[cH][cH][c](O)[c]2OC |
| 100 | FCC(C)C(C)C([c]1[cH][c]([c](O)[cH][cH]1)OC)[c]([cH]2)[cH][cH][c](O)[c]2OC |
| 101 | FCC(C)C(C)C([c]1[cH][c]([c](O)[cH][cH]1)OC)[c]([cH]2)[cH][cH][c](O)[c]2OC |
| 102 | O[c]([cH][cH]1)[c](OC)[cH][c]1C(C(C(C)C)CC)[c]([cH]2)[cH][cH][c](O)[c]2OC |
| 103 | O[c]([cH][cH]1)[c](OC)[cH][c]1C(C(C(C)C)CCCCC)[c]([cH]2)[cH][cH][c](O)[c]2OC |
| 104 | O[c]([cH][cH]1)[c](OC)[cH][c]1C([c]2[cH][cH][c]([c]([cH]2)OC)O)C(C(C)C)CCCCCCCC |
| 105 | O[c]([cH][cH]1)[c](OC)[cH][c]1C(C(C(C)C)CC(C)C)[c]([cH]2)[cH][cH][c](O)[c]2OC |
| 106 | CO[c]([cH][cH]1)[c](OC)[cH][c]1C2OC(C=C[c]3[cH][cH][cH][cH][c]3(OC))OCC2CC=C[c]4[c](OC)[cH][cH][cH][c]4OC |
| 107 | CO[c]1[cH][cH][cH][cH][c]1C=CC(OC2)OC([c]3[cH][cH][cH][cH][c]3(OC))C2CC=C[c]4[c](OC)[cH][cH][cH][c]4OC |
| 108 | CO[c]1[cH][cH][cH][cH][c]1C=CC(OC2)OC([c]3[c]([cH][cH][cH][c]3(OC))OC)C2CC=C[c]4[c](OC)[cH][cH][cH][c]4OC |
| 109 | CO[c]1[cH][cH][cH][cH][c]1C=CCC(CO2)C([c]3[cH][cH][cH][cH][c]3(OC))OC2C=C[c]4[cH][cH][cH][cH][c]4OC |
| 110 | CO[c]([cH][cH]1)[c](OC)[cH][c]1C2OC(C=C[c]3[cH][cH][cH][cH][c]3(OC))OCC2CC=C[c]4[c](OC)[cH][cH][cH][c]4OC |
| 111 | CO[c]1[cH][cH][cH][cH][c]1C=CC(OC2)OC([c]3[cH][cH][cH][cH][c]3(OC))C2CC=C[c]4[c](OC)[cH][cH][cH][c]4OC |
| 112 | CO[c]1[cH][cH][cH][cH][c]1C=CC(OC2)OC([c]3[c]([cH][cH][cH][c]3(OC))OC)C2CC=C[c]4[c](OC)[cH][cH][cH][c]4OC |
| 113 | CO[c]1[cH][cH][cH][cH][c]1C=CCC(CO2)C([c]3[cH][cH][cH][cH][c]3(OC))OC2C=C[c]4[cH][cH][cH][cH][c]4OC |
| 114 | O=C1C=C2OCOC2=CC1(CC=C)C(C)C[c]([cH]3)[cH][c](OC)[c](O)[c]3OC |
| 115 | CO[c]([cH]1)[c](OC)[c](OC)[cH][c]1C(C2(C))O[c]3[c]2[c]4OCO[c]4[cH][c]3CC=C |
| 116 | O[c]([c](OC)[cH]1)[c](OC)[cH][c]1C2O[c]3[c](OC)[cH][c](CCCO)[cH][c]3C2COC4OC(C)C(O)C(O)C4O |
| 117 | O[c]([c](OC)[cH]1)[c](OC)[cH][c]1C2O[c]3[c](OC)[cH][c](CCCO)[cH][c]3C2COC4OC(C)C(O)C(O)C4O |
| 118 | O=C(C=C[c]1[cH][c]([c](O)[cH][cH]1)OC)OCCC[c]([cH][cH]2)[cH][c](O)[c]2OC(C([c]3[cH][cH][c]([c]([cH]3)OC)O)O)COC(=O)C=C[c]([cH]4)[cH][cH][c](O)[c]4OC |
| 119 | O=C(C=C[c]1[cH][c]([c](O)[cH][cH]1)OC)OCCC[c]([cH][cH]2)[cH][c](OC)[c]2OC(C([c]3[cH][cH][c]([c]([cH]3)OC)O)O)COC(=O)C=C[c]([cH]4)[cH][cH][c](O)[c]4OC |
| 120 | O[c]([cH][cH]1)[c](OC)[cH][c]1C(C2(O)(CO))OCC2C[c]([cH]3)[cH][cH][c](O)[c]3OC |
| 121 | O=C(O)C1C(C)C[c]2[cH][c]3OCO[c]3[cH][c]2C1[c]([cH]4)[cH][c]5OCO[c]5[c]4OC |
| 122 | O=C(C21)OCC2C[c]3[c](OC)[c]4OCO[c]4[cH][c]3C1[c]([cH]5)[cH][c]6OCO[c]6[c]5OC |
| 123 | O=C(OCC)C=C[c]([cH]1)[cH][c](O)[c](O2)[c]1C(C(OCC)=O)C2[c]3[cH][c](O)[c](O)[cH][cH]3 |
| 124 | OC(C1)C(=O)C2(O)C(O)C1(CC=C)C(C)C2C3CCC4OCOC4C3 |
| 125 | OC=1C(OC)=C(OC)CC(CCC2(C))C=1C3C(OC)C4OCOC4C=C3C2OC(=O)C(C)=CC |
| 126 | O=C(C)C(C1(C(=O)OCC))=C[c]2[cH][c](O)[c](O)[cH][c]2C1[c]3[cH][c](O)[c](O)[cH][cH]3 |
| 127 | O=C(O)C(C1(C(=O)OCC))=C[c]2[cH][c](O)[c](O)[cH][c]2C1[c]3[cH][c](O)[c](O)[cH][cH]3 |
| 128 | O=C(OCC)[c]([c]1(C(=O)O))[cH][c]2[cH][c](O)[c](O)[cH][c]2[c]1[c]3[cH][c](O)[c](O)[cH][cH]3 |
| 129 | O[c]([c](O)[cH]1)[cH][cH][c]1C(C2)[c]3[cH][c](O)[c](O)[cH][c]3C=C2C(=O)OCC |
| 130 | O=C(OC(C(=O)OC)C[c]1[cH][c](O)[c](O)[cH][cH]1)[c]([cH]2)[cH][c]3[cH][c](O)[c](O)[cH][c]3[c]2[c]4[cH][c](O)[c](O)[cH][cH]4 |
| 131 | OCC1C(=O)OCC1C([c]2[cH][cH][c](OC)[c](OC)[cH]2)[c]([cH]3)[cH][cH][c](O)[c]3OC |
| 132 | OCC1C(=O)OCC1C([c]2[cH][cH][c](O)[c](OC)[cH]2)C(C3)=CC=C(O)C3OC |
| 133 | O[c]([c](OC)[cH]1)[c](OC)[cH][c]1[c](o2)[c](CO)[c]([cH]3)[c]2[c](OC)[cH][c]3C=CCOC |
| 134 | O[c]([cH][cH]1)[cH][cH][c]1C(O2)C(C)[c]([cH]3)[c]2[cH][cH][c]3C=CCO |
| 135 | OCC1[c]2[cH][c](C=CCOC)[cH][cH][c]2OC1[c]3[cH][cH][c](O)[cH][cH]3 |
| 136 | O=CC=C[c]([cH]1)[cH][c](OC)[c](O2)[c]1C(CO)C2[c]3[cH][cH][c](O)[cH][cH]3 |
| 137 | O[c]([cH][cH]1)[c](OC)[cH][c]1[c](o2)[c](OC)[c]([cH]3)[c]2[c](OC)[cH][c]3C(O)C(O)CO |
| 138 | OCC(C[c]1[cH][c](OC)[c](OC)[c](OC)[cH]1)C(COC(=O)C)C[c]2[cH][cH][c]3OCO[c]3[cH]2 |
| 139 | O[c]([cH][cH]1)[c](O)[cH][c]1C2OCC3C2COC3[c]4[cH][c](O)[c](O)[cH][cH]4 |
| 140 | O[c]([cH]1)[c](O)[cH][cH][c]1C2OCC3C2COC3[c]4[cH][c](O)[c](O)[cH][cH]4 |
| 141 | O[c]([cH][cH]1)[cH][cH][c]1C=CCC23CCN(C)C3N[c]4[c]2[cH][cH][cH][cH]4 |
| 142 | O[c]([c](OC)[cH]1)[c](OC)[cH][c]1C(C2COC(=O)C2(=C))[c]([cH]3)[cH][c](OC)[c](OC)[c]3OC |
| 143 | O[c]([cH]1)[c](OC)[c](OC)[cH][c]1C(C2COC(=O)C2(=C))[c]([cH]3)[cH][c](OC)[c](OC)[c]3OC |
| 144 | O[c]([cH]1)[c](OC)[c](OC)[cH][c]1C(C2COC(=O)C2(C))[c]([cH]3)[cH][c]4OCO[c]4[c]3O |
| 145 | O[c]([cH]1)[c](OC)[c](O)[cH][c]1C(C2COC(=O)C2(C))[c]([cH]3)[cH][c]4OCO[c]4[c]3OC |
| 146 | O[c]([c](OC)[cH]1)[c](OC)[cH][c]1C(C2COC(=O)C2(CO))[c]([cH]3)[cH][c]4OCO[c]4[c]3OC |
| 147 | OC([c]1[cH][cH][c](O)[c](OC)[cH]1)C(CO)O[c]([c]2(O))[cH][c](OC)[c](O)[c]2C=CC=O |
| 148 | O[c]([cH][cH]1)[c](OC)[cH][c]1C2O[c]3[c](OC)[cH][c](OC)[cH][c]3C2CO |
| 149 | O[c]([cH]1)[cH][c](OC)[cH][c]1C(O2)C(CO)[c]([cH]3)[c]2[c](OC)[cH][c]3C=CCO |
| 150 | O[C](=C1)=CC(OC)=CC1C(O2)C(CO)C(C3)=C2C(OC)=CC3=[CH]=CCOC |
| 151 | O[c]([c](OC)[cH]1)[cH][cH][c]1C(OC)C(C2(CO))COC2[c]([cH]3)[cH][cH][c](O)[c]3OC |
| 152 | O=C(NCC[c]1[cH][cH][c](O)[cH][cH]1)C=C[c]([cH]2)[cH][c](OC)[c](O3)[c]2C(CO)C3[c]([cH]4)[cH][c](O)[cH][c]4OC |
| 153 | O=C(NCC[c]1[cH][cH][c](O)[cH][cH]1)C=C[c]([cH]2)[cH][c](OC)[c](O3)[c]2C(CO)C3[c]([cH]4)[cH][c](OC)[c](O)[c]4OC |
| 154 | O=C(NCC[c]1[cH][cH][c](O)[cH][cH]1)CC[c]([cH]2)[cH][c](CC)[c](O3)[c]2C(CO)C3[c]([cH]4)[cH][c](O)[cH][c]4OC |
| 155 | O=C(NCC[c]1[cH][cH][c](O)[cH][cH]1)CC[c]([cH]2)[cH][c](CC)[c](O3)[c]2C(CO)C3[c]([cH]4)[cH][c](OC)[c](O)[c]4OC |
| 156 | OCC(C[c]1[cH][c](OC)[c](O)[cH][cH]1)C(CO)C[c]([cH]2)[cH][cH][c](O)[c]2OC |
| 157 | OCC1C(C[c]2[cH][cH][c](O)[c](OC)[cH]2)COC1[c]([cH][c]3(OC))[cH][cH][c]3OC4OC(CO)C(O)C(O)C4O |
| 158 | O[c]([c](OC)[cH]1)[cH][cH][c]1C2OCC3C2COC3[c]([cH][cH]4)[cH][c](OC)[c]4OC(O5)C(O)C(O)C(O)C5CO |
| 159 | O[c]([cH][cH]1)[c](OC)[cH][c]1C2OCC3C2COC3[c]([cH][cH]4)[cH][c](OC)[c]4OC5OC(CO)C(O)C(O)C5OC(C6(O))OCC6(O)CO |

Figure S1: ROC curves generated from the RF models, for each studied enzyme.

Cross-validation

**JNK-3**

Test


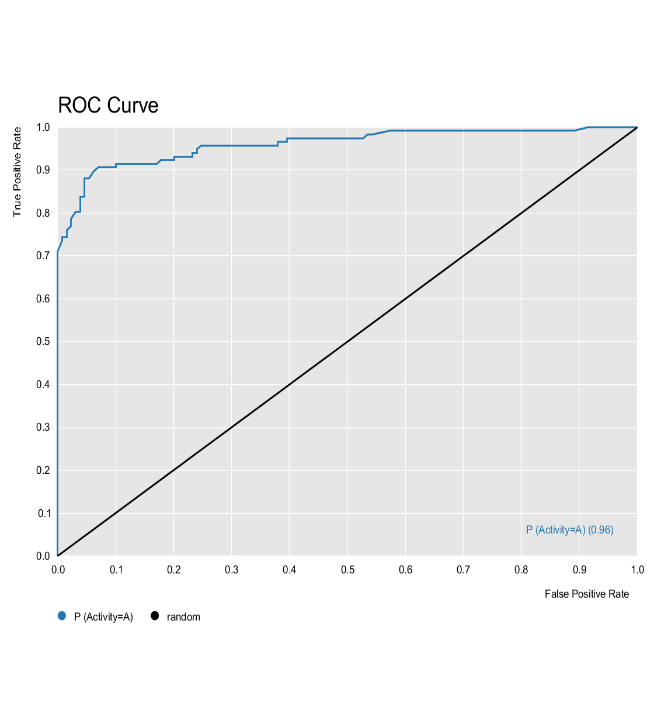

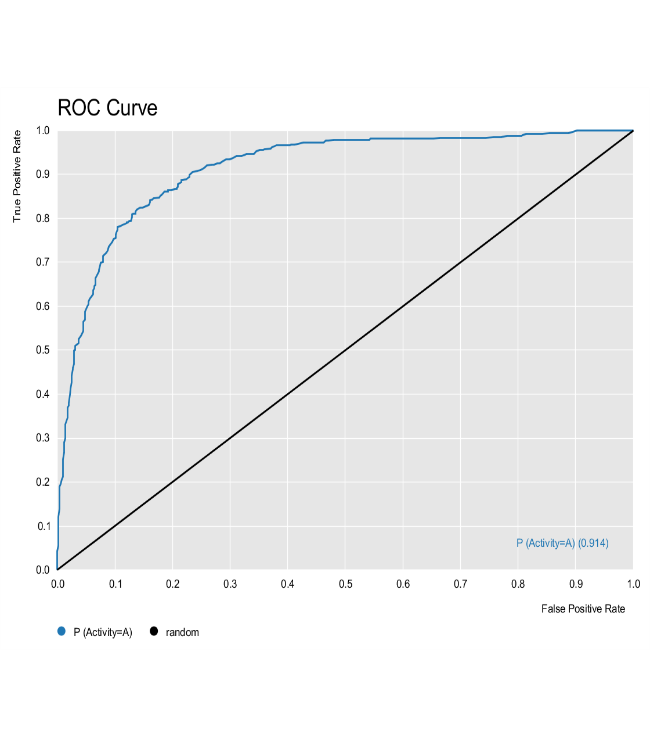


**PTP1B**

Cross-validation

Test


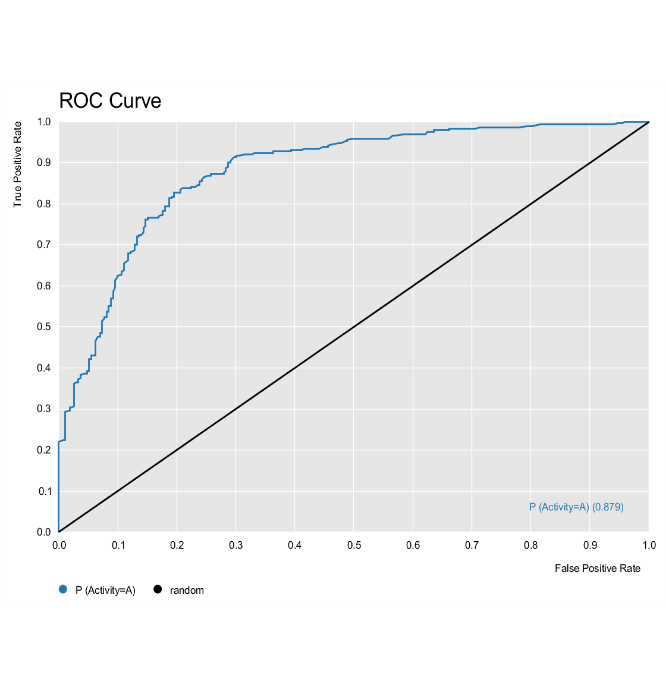

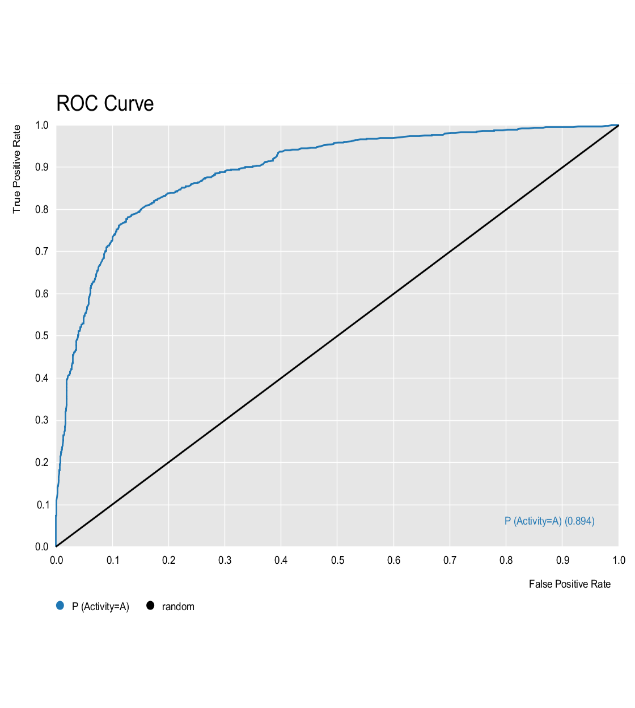


**NFR2**

Cross-validation

Test


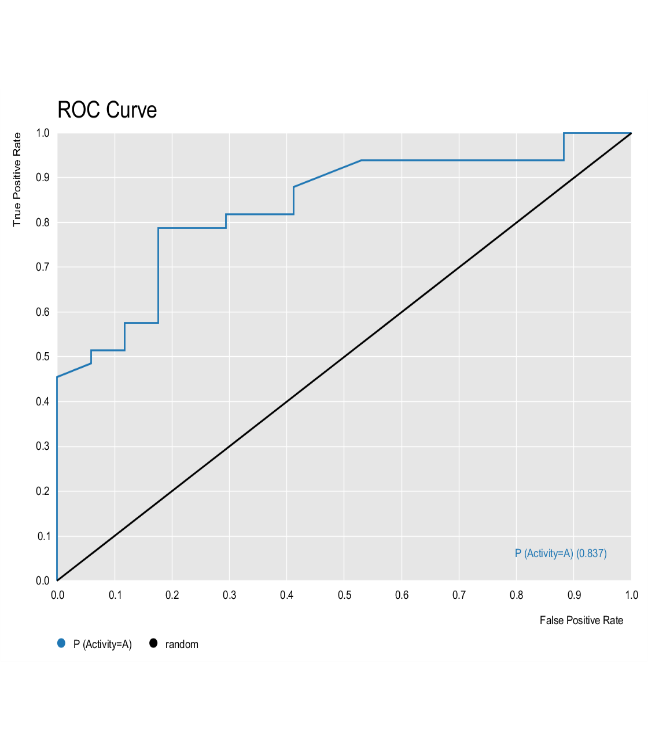

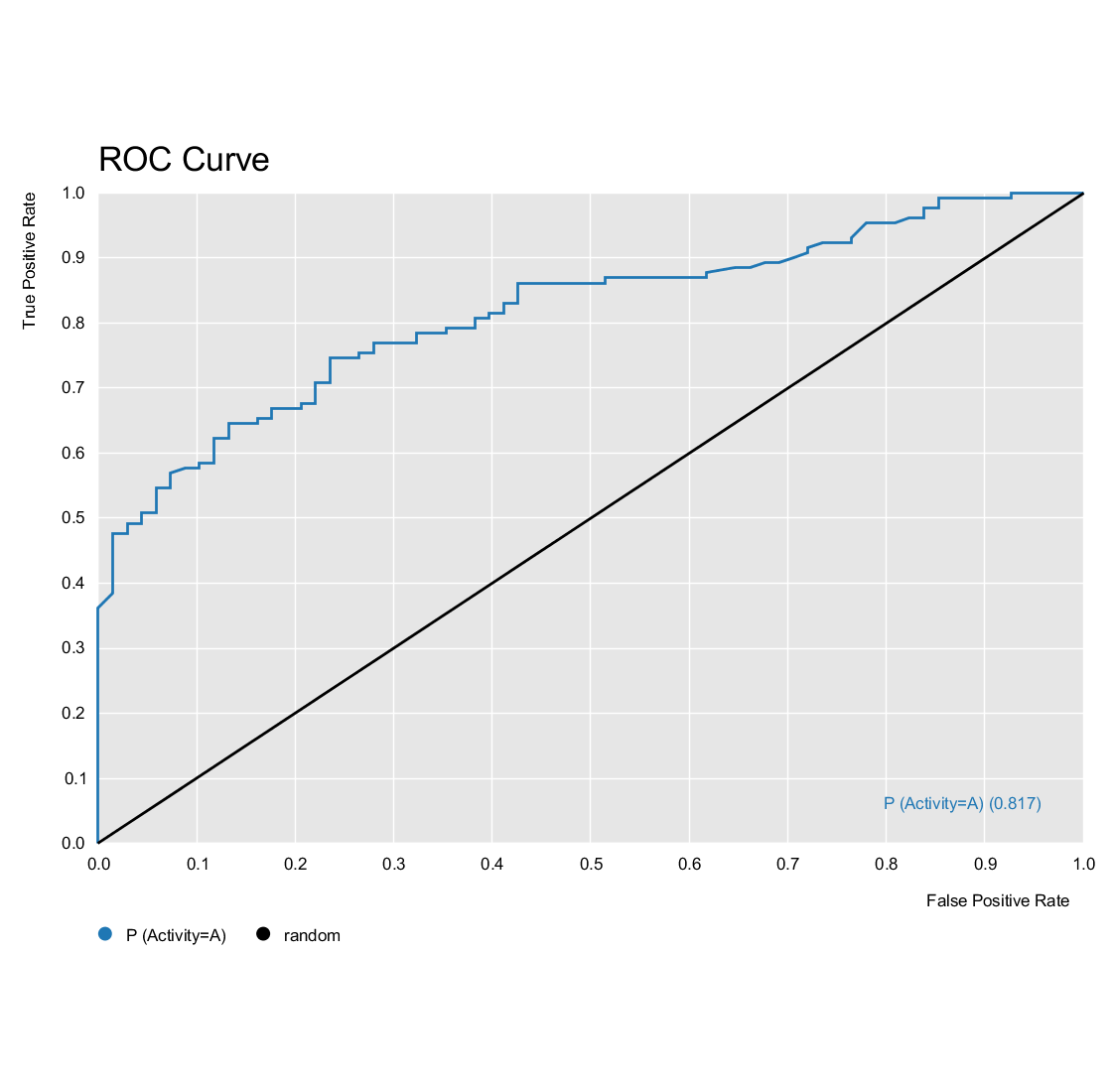


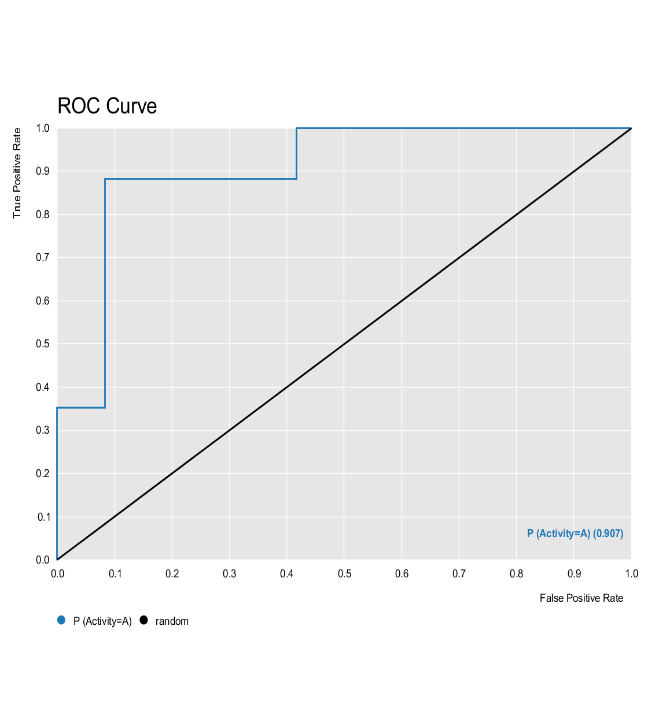

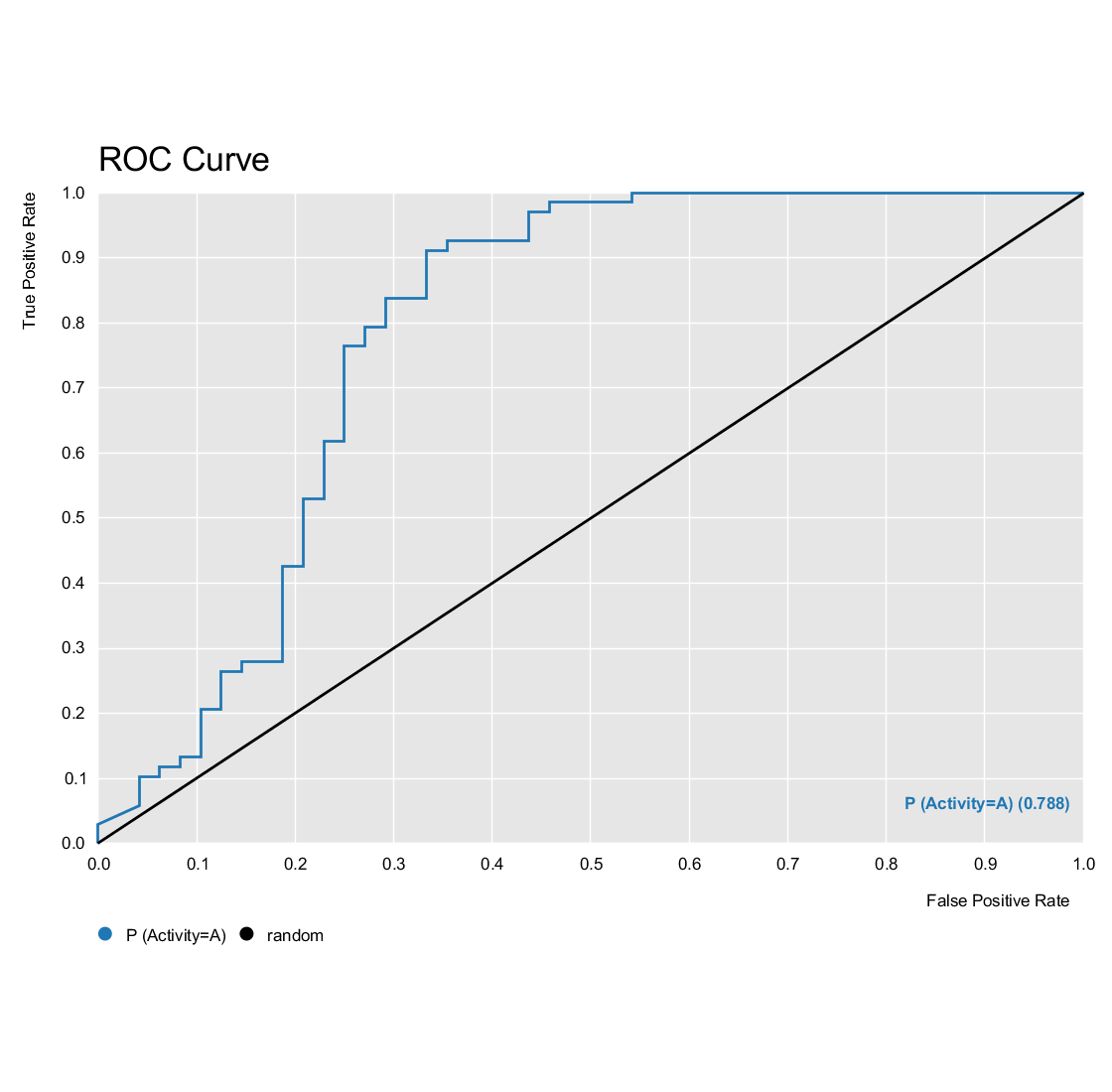


**PDE5**

Cross-validation

**NOX1**

Test

Cross-validation

Test


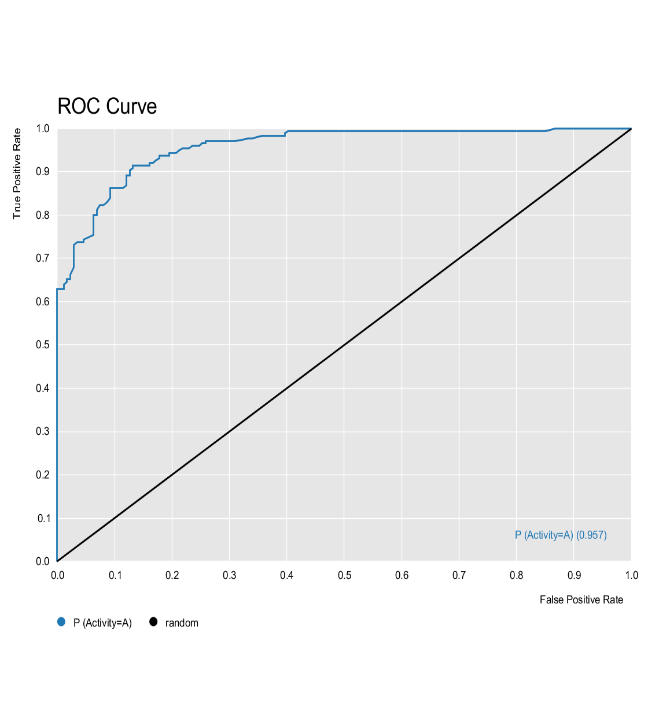

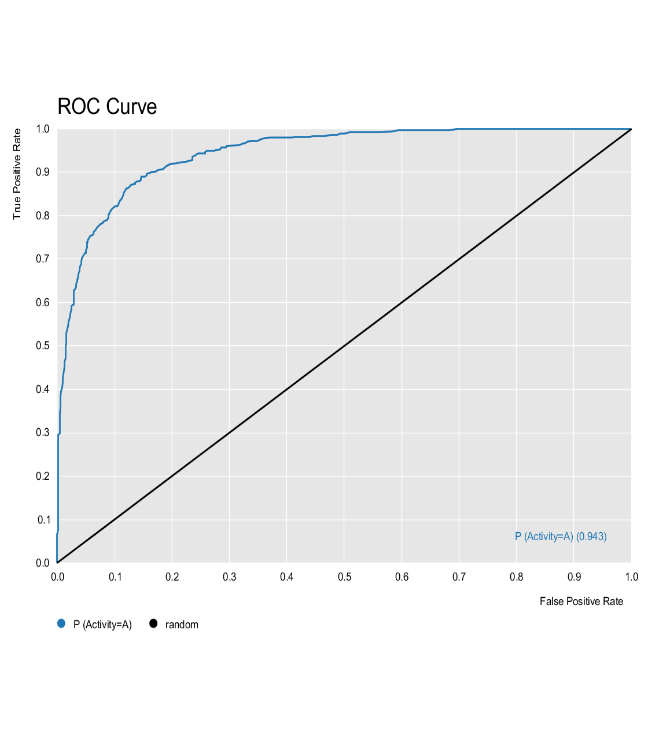


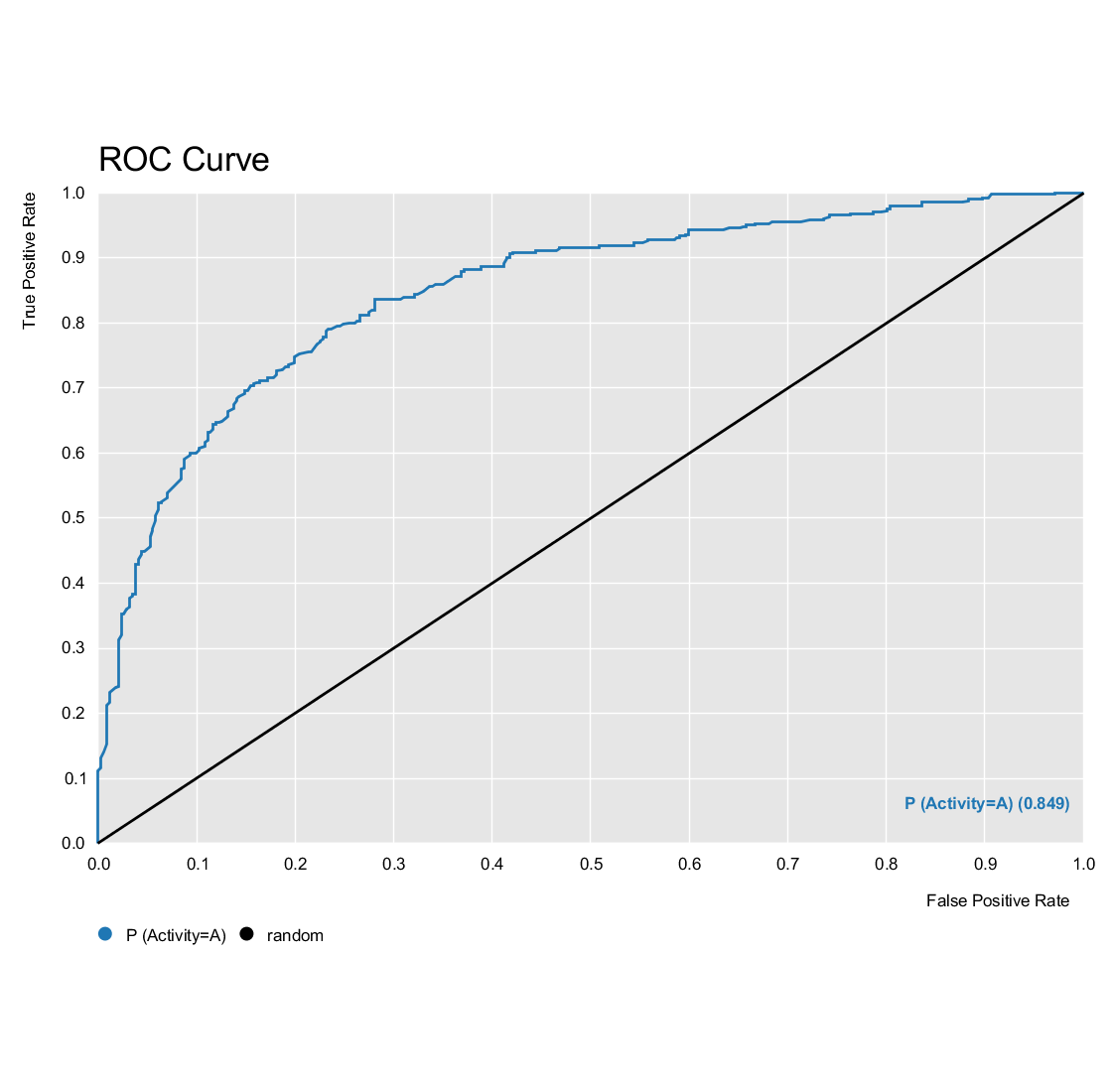

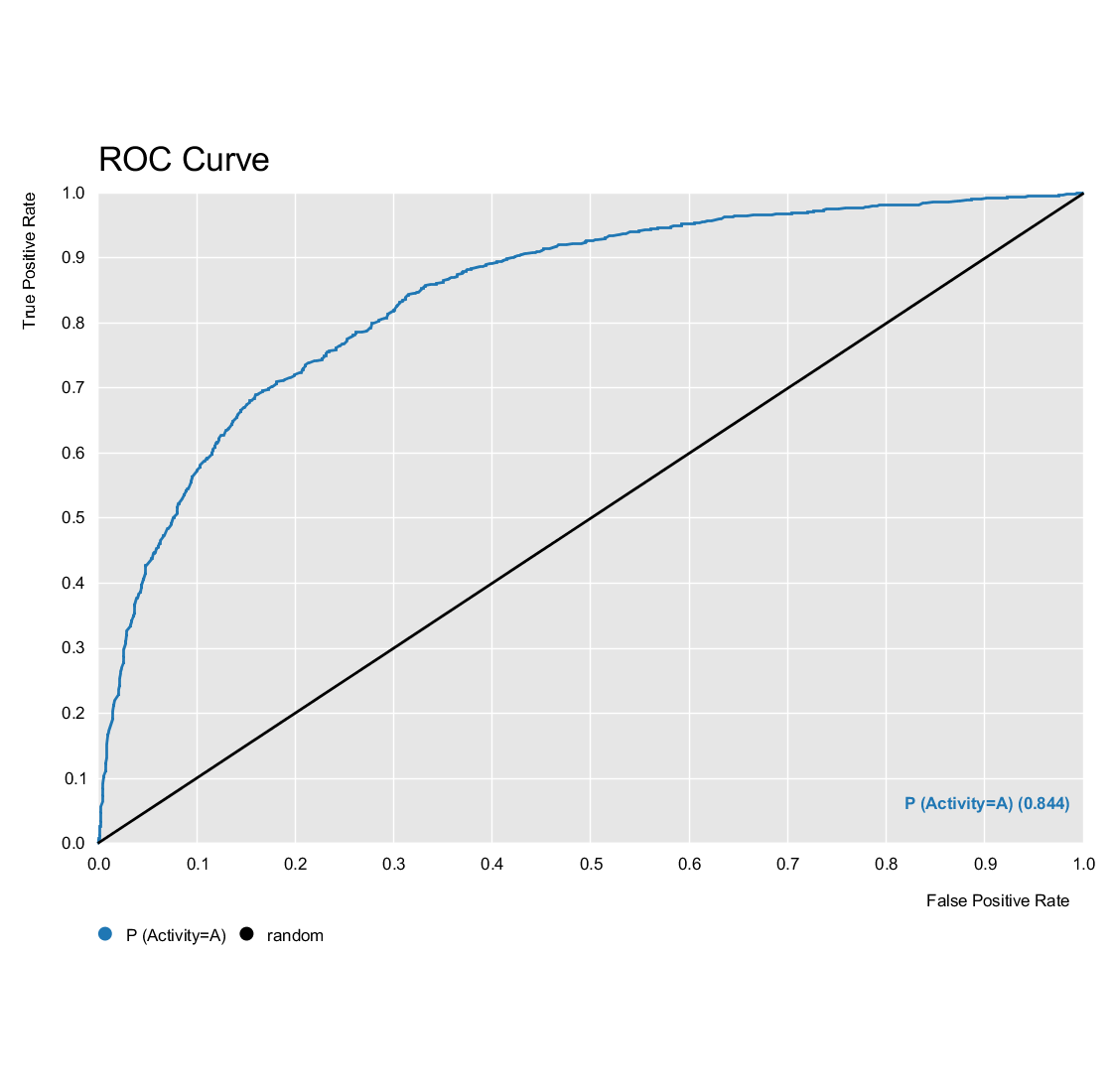


Cross-validation

Test

**COX2**


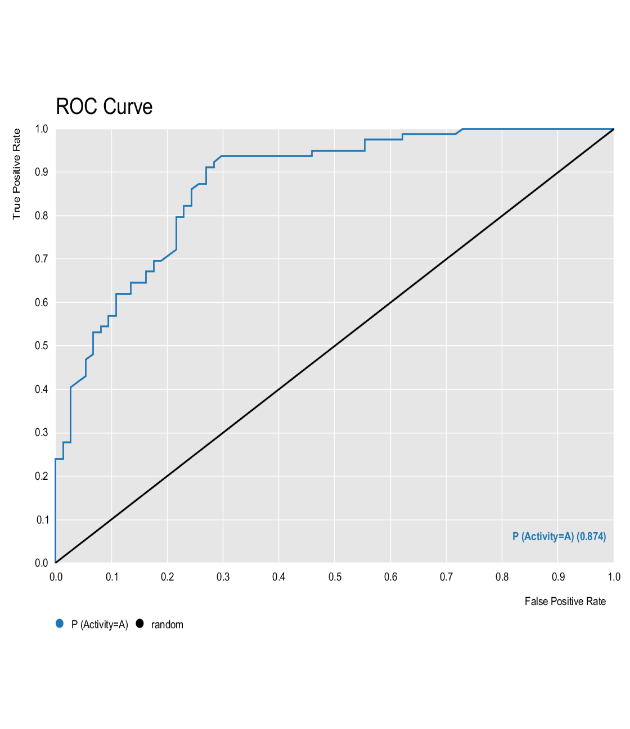

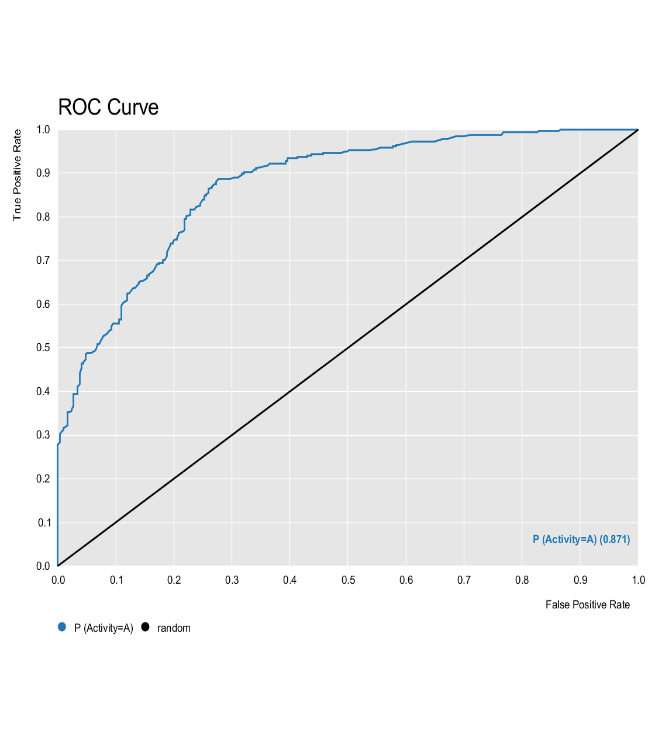


Cross-validation

***i*NOS**

Test

Table S2: The physical and chemical properties of lignans that are considered to be potentially active against Alzheimer's disease and have multitargeting effects against four or more enzymes.

| ID | MW | TPSA | Score de biodisponibilidade | Violações de Lipinski | Log P Consensus |
| --- | --- | --- | --- | --- | --- |
| 6 | 328.4 | 47.92 | 0.55 | 1 | 4.25 |
| 11 | 284.35 | 49.69 | 0.55 | 0 | 3.11 |
| 19 | 358.43 | 57.15 | 0.55 | 0 | 3.73 |
| 33 | 416.51 | 77.38 | 0.55 | 0 | 4.07 |
| 36 | 556.56 | 153.37 | 0.17 | 2 | 2.93 |
| 38 | 554.54 | 157.28 | 0.17 | 2 | 3.27 |
| 39 | 674.65 | 176.51 | 0.17 | 5 | 4.49 |
| 41 | 676.66 | 179.67 | 0.17 | 5 | 4.26 |
| 42 | 646.64 | 170.44 | 0.17 | 5 | 4.36 |
| 51 | 526.49 | 168.28 | 0.17 | 2 | 2.54 |
| 52 | 384.34 | 100.52 | 0.55 | 0 | 2.07 |
| 54 | 442.42 | 106.59 | 0.55 | 0 | 2.68 |
| 64 | 416.46 | 64.61 | 0.55 | 0 | 3.7 |
| 69 | 564.67 | 99.38 | 0.55 | 1 | 6.3 |
| 71 | 280.32 | 46.53 | 0.55 | 0 | 3.76 |
| 106 | 372.5 | 58.92 | 0.55 | 1 | 4.98 |
| 107 | 532.62 | 64.61 | 0.55 | 1 | 5.48 |
| 116 | 384.42 | 55.38 | 0.55 | 0 | 4.04 |
| 117 | 536.57 | 156.53 | 0.17 | 2 | 1.38 |
| 118 | 536.57 | 156.53 | 0.17 | 2 | 1.46 |
| 119 | 716.73 | 190.67 | 0.17 | 5 | 4.7 |
| 123 | 412.39 | 81.68 | 0.55 | 0 | 2.99 |
| 129 | 342.34 | 107.22 | 0.55 | 0 | 2.43 |
| 130 | 506.46 | 173.98 | 0.17 | 2 | 3.14 |
| 134 | 282.33 | 49.69 | 0.55 | 0 | 3.06 |
| 135 | 312.36 | 58.92 | 0.55 | 0 | 2.75 |
| 136 | 326.34 | 75.99 | 0.55 | 0 | 2.33 |
| 142 | 430.45 | 92.68 | 0.55 | 0 | 3.19 |
| 146 | 432.42 | 112.91 | 0.55 | 0 | 2.36 |
| 153 | 521.56 | 126.71 | 0.55 | 1 | 3.21 |
| 154 | 491.58 | 108.25 | 0.55 | 0 | 3.87 |
| 155 | 521.6 | 117.48 | 0.55 | 1 | 3.86 |
| 158 | 520.53 | 156.53 | 0.17 | 2 | 0.59 |
| 159 | 652.64 | 215.45 | 0.17 | 3 | -0.43 |

Table S3: Pharmacokinetic properties of the lignans that are considered to be potentially active against Alzheimer's disease and have multitargeting effects against four or more enzymes. Lignans that are predicted to cross the blood-brain barrier are highlighted in bold.

| ID | GI^1^ | BBB^2^ | Pgp^3^ | CYP1A2 | CYP2C19 | CYP2C9 | CYP2D6 | CYP3A4 |
| --- | --- | --- | --- | --- | --- | --- | --- | --- |
| 6 | **High** | **Yes** | **No** | **No** | **Yes** | **Yes** | **Yes** | **No** |
| 11 | **High** | **Yes** | **Yes** | **Yes** | **Yes** | **No** | **Yes** | **No** |
| 19 | **High** | **Yes** | **Yes** | **No** | **No** | **No** | **Yes** | **Yes** |
| 33 | High | No | No | No | No | No | Yes | Yes |
| 36 | Low | No | No | No | No | No | No | Yes |
| 38 | Low | No | No | No | No | Yes | No | Yes |
| 39 | Low | No | No | No | Yes | Yes | No | No |
| 41 | Low | No | Yes | No | No | Yes | No | No |
| 42 | Low | No | No | No | No | Yes | No | No |
| 51 | Low | No | No | No | No | Yes | No | Yes |
| 52 | High | No | No | No | No | Yes | Yes | Yes |
| 54 | High | No | No | No | No | Yes | No | Yes |
| 64 | **High** | **Yes** | **No** | **No** | **No** | **No** | **Yes** | **No** |
| 69 | Low | No | No | No | No | No | Yes | No |
| 71 | **High** | **Yes** | **No** | **Yes** | **Yes** | **Yes** | **Yes** | **Yes** |
| 106 | High | No | Yes | No | No | No | Yes | No |
| 107 | High | No | Yes | No | Yes | No | No | No |
| 116 | **High** | **Yes** | **No** | **No** | **Yes** | **Yes** | **Yes** | **Yes** |
| 117 | Low | No | Yes | No | No | No | No | No |
| 118 | Low | No | Yes | No | No | No | No | No |
| 119 | Low | No | No | No | No | Yes | No | No |
| 123 | High | No | No | No | Yes | Yes | Yes | Yes |
| 129 | High | No | Yes | No | No | No | Yes | No |
| 130 | Low | No | No | No | No | Yes | No | No |
| 134 | **High** | **Yes** | **Yes** | **Yes** | **No** | **No** | **Yes** | **No** |
| 135 | **High** | **Yes** | **Yes** | **No** | **No** | **No** | **Yes** | **No** |
| 136 | **High** | **Yes** | **Yes** | **No** | **No** | **No** | **No** | **No** |
| 142 | High | No | No | No | Yes | Yes | Yes | Yes |
| 146 | High | No | No | No | No | Yes | Yes | Yes |
| 153 | High | No | Yes | No | Yes | Yes | Yes | Yes |
| 154 | High | No | Yes | No | Yes | No | Yes | Yes |
| 155 | High | No | Yes | No | Yes | No | Yes | Yes |

^1^GI: Gastrointestinal absorption

^2^BBB: Blood-brain barrier

^3^P-gp: P-glycoprotein

Table S4: Toxicity evaluations for lignans with the best ADMET profiles, that are potentially active and that have multitarget effects against four or more enzymes. Lignans that did not present toxicity for any of the analyzed parameters are highlighted in bold.

| ID | Mutagenic | Tumorogenic | Reproductive effect | Irritant |
| --- | --- | --- | --- | --- |
| 6 | **No** | **No** | **No** | **No** |
| 11 | **No** | **No** | **No** | **No** |
| 19 | **No** | **No** | **No** | **No** |
| 64 | **No** | **No** | **No** | **No** |
| 71 | Low | High | No | High |
| 116 | **No** | **No** | **No** | **No** |
| 134 | No | No | No | High |
| 135 | **No** | **No** | **No** | **No** |
| 136 | Low | High | No | Low |
